# Protocol for PI3K inhibitor-free differentiation and maturation of human iPSC-derived arterial- and venous-like endothelial cells

**DOI:** 10.1016/j.xpro.2026.104668

**Published:** 2026-07-30

**Authors:** Oliwia N. Mruk, Alexandra J. Musk, Ralitsa R. Madsen

**Affiliations:** 1 MRC Protein Phosphorylation and Ubiquitylation Unit, School of Life Sciences, University of Dundee, Dundee DD1 5EH, UK

**Keywords:** Cell Biology, Cell culture, Cell isolation, Developmental biology, Stem Cells, Cell Differentiation

## Abstract

Vascular malformations caused by genetic PI3K pathway activation are highly debilitating and hard to treat. Here, we present a PI3K inhibitor-free protocol to differentiate arterial and venous endothelial cells from human induced pluripotent stem cells (iPSCs) in 2D and 3D formats. We describe steps for iPSC culture, cell seeding on a synthetic matrix, defined media preparation, magnetic-activated cell sorting, and endothelial cell maturation under shear stress. We then detail procedures for determining cell density and culture duration for robust functional phenotyping.

## Highlights

• Differentiate iPSCs into arterial and venous endothelial cells without PI3K inhibition
• Generate venous endothelial cells efficiently using a 3D scaffold-free format
• Achieve high endothelial cell purity by magnetic-activated cell sorting for VE-cadherin
• Expose iPSC-derived endothelial cells to shear stress for longer phenotypic stability

## Abstract (editor highlights)

Publisher’s note: Undertaking any experimental protocol requires adherence to local institutional guidelines for laboratory safety and ethics.

## Graphical abstract

**Figure:**
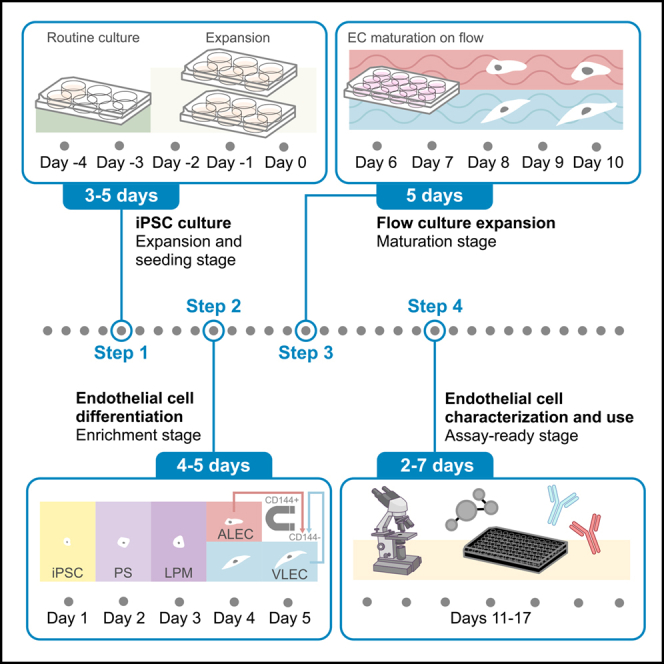

## Teaser

Vascular malformations caused by genetic PI3K pathway activation are highly debilitating and hard to treat. Here, we present a PI3K inhibitor-free protocol to differentiate arterial and venous endothelial cells from human induced pluripotent stem cells (iPSCs) in 2D and 3D formats. We describe steps for iPSC culture, cell seeding on a synthetic matrix, defined media preparation, magnetic-activating cell sorting, and endothelial cell maturation under shear stress. We then detail procedures for cell density and culture duration for robust functional phenotyping.

## Before you begin

Mosaic activating mutations in *PIK3CA*, the gene encoding the catalytic subunit of phosphoinositide 3-kinase alpha (PI3Kα), cause 25%–30% of congenital venous malformations (VMs),^1^ the most common type of vascular malformation. Although exceedingly rare, strongly activating *PIK3CA* mutations have also been reported in arteriovenous malformations (AVMs) in the context of complex overgrowth syndromes collectively termed *PIK3CA*-related overgrowth spectrum (PROS).^2^^,^^3^^,^^4^ Notably, AVMs are more common in the related *PTEN* hamartoma tumor syndrome (PHTS), a group of disorders caused by germline loss-of-function mutations in the tumor suppressor *PTEN*.^5^^,^^6^^,^^7^^,^^8^ The latter dephosphorylates the PI(3,4,5)P_3_ second messenger produced by PI3Kα; accordingly, PHTS and PROS share partially overlapping clinical findings. Better understanding of these PI3K-driven vascular pathologies, including the evidence for apparent developmental lineage skewing, and identification of improved treatment options requires access to high-fidelity, human “disease-in-a-dish”-based preclinical model systems. Several independent workflows for differentiation of human pluripotent stem cells (hPSC) into either arterial- (ALECs) or venous-like endothelial cells (VLECs) already exist, but vary substantially with respect to duration, choice of differentiation components, extracellular matrix, and lineage-specific marker evaluation.^9^^,^^10^^,^^11^^,^^12^ Amongst these, the recent protocol by Ang/Loh et al. offers one of the most time-efficient and extensively benchmarked solutions, leading to robust VLEC and ALEC generation within 4–5 days.^10^^,^^13^ Crucially, however, it features the use of GDC0941, a class I PI3K inhibitor, which inhibits all p110 catalytic isoforms at the specified concentration of 2.5 μM. Thus, despite its robustness, the existing workflow would not be compatible with the modelling of developmental, PI3K-driven vascular malformations.

Building on Ang and Loh et al.’s work, we present a robust PI3K inhibitor-free differentiation workflow for iPSC-derived ALEC and VLEC differentiation. We achieve this through the combined removal of insulin and GDC0941 at steps where both were previously required. Furthermore, we replace the use of Matrigel/Geltrex with synthetic laminin-511 E8 fragment as a more physiologically-relevant extracellular matrix,^14^^,^^15^^,^^16^ thereby facilitating fully defined differentiation culture conditions. A key innovation of our protocol is the option to initiate differentiation using scaffold-free iPSC spheroids, which substantially enhances VLEC generation. Lastly, we present extensively optimised post-differentiation culture conditions for robust ALEC and VLEC maturation, including critical parameters such as cell density and flow.

This protocol was initially optimised using the male WTC11 iPSC line and its CRISPR-derivative with mEGFP-tagged VE-Cadherin (AICS-0126-068:WTC-mEGFP-CDH5-cl68). It has subsequently been validated in multiple human female and male iPSC lines, including patient- and CRISPR-derived clones harbouring activating *PIK3CA* mutations. We note that the genetic background of each iPSC line may impact differentiation efficiency and outline key considerations to mitigate such variability.

### Innovation

This STAR protocol enables a PI3K inhibitor-free differentiation workflow for generating arterial and venous endothelial cells from human iPSCs in both two- (2D) and scaffold-free, three-dimensional (3D) culture formats. Building on an initial 2D differentiation protocol from Ang/Loh et al.,^10^^,^^13^ our method bypasses pharmacological PI3K pathway modulation and the use of undefined differentiation reagents such as Matrigel. A further innovation is the incorporation of a validated maturation step under shear stress once the endothelial cells have been generated. The protocol also provides critical parameters for cell density before, during, and after differentiation – all of which are essential for endothelial differentiation efficiency and phenotypic fidelity. Together, these advances establish a reproducible, high-fidelity platform that will be of interest to developmental biologists, bioengineers, as well as researchers seeking to model developmental vascular lineage skewing and congenital (arterio)venous malformations driven by genetic PI3K pathway activation, including *PIK3CA*-related overgrowth spectrum (PROS) and PTEN hamartoma tumor syndrome (PHTS).

### Institutional permissions

All work described in this protocol was conducted using previously established human iPSC lines under the appropriate institutional approvals. No iPSC reprogramming from primary human material was performed as part of this work.

Users intending to derive new iPSC lines or use the protocol with human embryonic stem cells (hESCs) must ensure that all necessary ethical approvals and regulatory permissions are obtained in accordance with their local institutional and national requirements.

### Coating plates with ECMatrix-511 silk E8 laminin substrate

**Timing: Variable (1–24 h; 15 min hands-on)**

Induced pluripotent stem cells (iPSCs) and the differentiated cells resulting from this workflow are cultured on tissue culture-treated polystyrene plates (Nunclon Delta) coated with ECMatrix-511 Silk E8 Laminin Substrate, as listed in the key resources table. For this workflow, we describe initial culture and differentiation in a 6-well plate format, followed by expansion and maturation of pure endothelial cells in a 12-well plate format. The following procedure should be performed in a biosafety cabinet following appropriate aseptic technique.

1. Dilute the ECMatrix-511 Silk E8 Laminin Substrate stock from a 0.5 mg/mL stock solution with sterile, ideally cold Dulbecco’s Phosphate-Buffered Saline (DPBS; no calcium, no magnesium) to achieve a 2.5 μg/mL working solution.
  a. Ensure immediate and sufficient mixing without vortexing.

***Note:*** The final Laminin Substrate amount should correspond to 0.25 μg/cm^2^ and cover the well fully.

2. Use 1 mL and 500 μL of the working solution to coat each well in a 6-well and 12-well plate, respectively.
3. Gently move the plate in a “North-South-East-West” motion to ensure complete coverage of the bottom surface.

**Figure 1:**
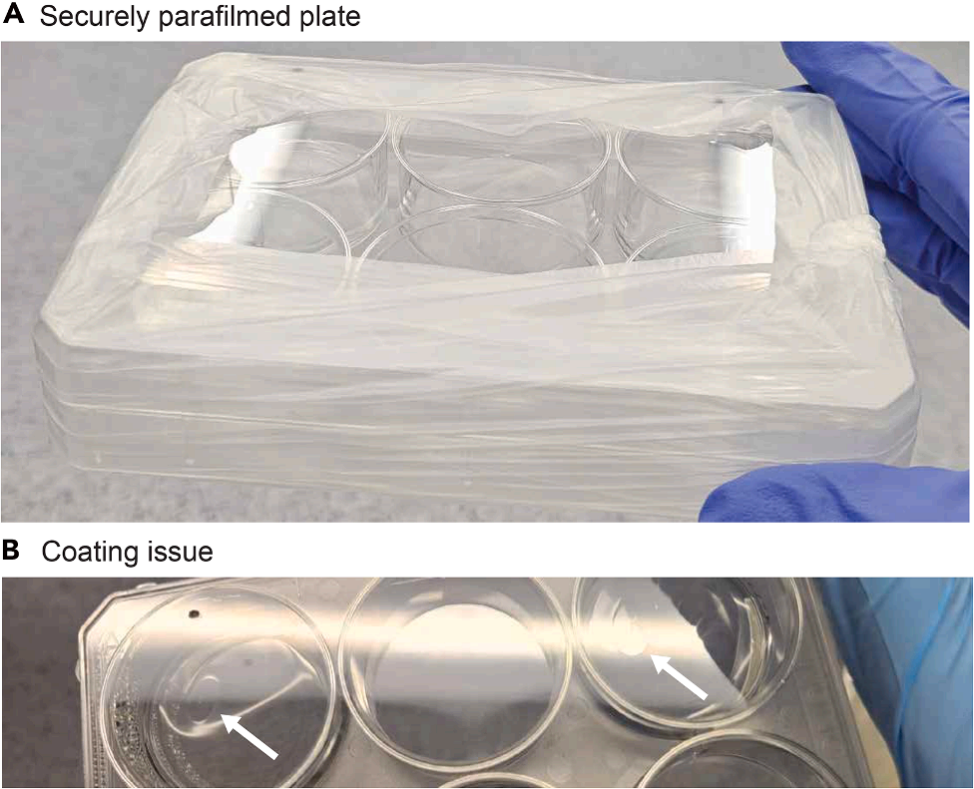
ECMatrix-511 Silk E8 Laminin Substrate coating and storage of plates (A) Securely parafilmed plate, ready for longer-term storage at 4°C. (B) Issue with coating; white arrows point to areas of the plate where the coating dried out and, therefore, cells will not attach.

**CRITICAL:** If any areas are not covered by ECMatrix (see Figure 1B for an example of uneven coating leading to uneven drying), cells will fail to attach.

4. Incubate the plates at 37°C for 1 h.
  a. Alternatively, leave the plates at 19°C–23°C for 3 h or at 4°C for at least 20 h (Parafilmed).

**CRITICAL:** Do not leave ECMatrix-511 Silk E8 Laminin Substrate-coated plates at 37°C for more than 24 h. This increases the risk of the coating drying out (Figure 1B), which will critically impact iPSC adherence, survival, and differentiation. Topping up the coated wells with extra DPBS will have a similar effect due to dilution of the matrix and should be avoided.

***Optional:*** Plates can be batch-coated in advance, secured with Parafilm, and stored at 4°C for up to 14 days, provided the coating is even and has not evaporated across all or some of the well surface (Figure 1). Keep the plates on a level surface to avoid uneven coating. When ready to use, pre-warm the plates to 19°C–23°C for 30 min.

### Thawing iPSCs

**Timing: 20 min**

Here, we describe a routine iPSC thawing procedure for subsequent maintenance culture in StemFlex Medium and on ECMatrix-511 Silk E8 Laminin Substrate. StemFlex Medium refers to the StemFlex Basal Medium supplemented with the StemFlex Supplement at 1X, according to the manufacturer’s instructions. The following procedure should be performed in a biosafety cabinet following appropriate aseptic technique.

**CRITICAL:** We recommend antibiotic-free iPSC culture to minimise any adverse effects of antibiotics on cell metabolism during growth. Extra care must therefore be taken to minimise the risk of contamination. This includes consistent use of filter tips, medium aliquots, and immediate closing of tubes/plates after each use.

**Figure 2:**
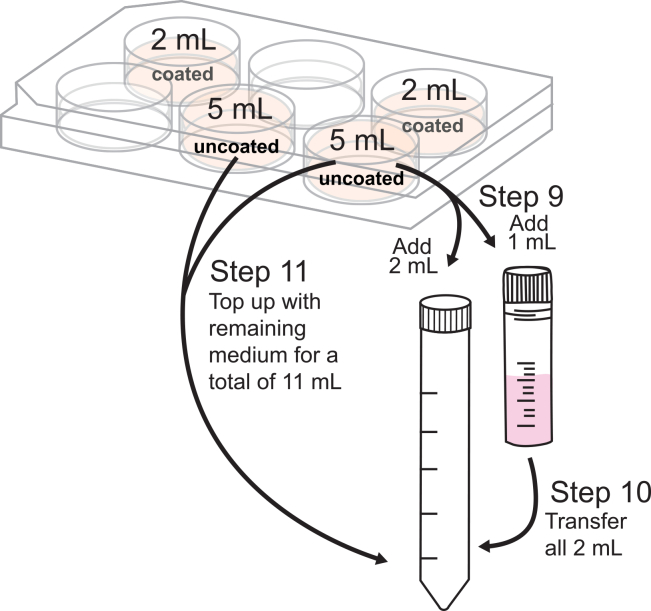
Schematic illustrating the procedure for Steps 9–11 during medium and cell transfer for thawing

5. Prepare in advance:
  a. The required volume of StemFlex Medium supplemented with RevitaCell Supplement diluted to 1X.
    ***Note:*** RevitaCell Supplement (containing a Rho-associated, coiled-coil containing protein kinase (ROCK) inhibitor and antioxidants) prevents stress-induced apoptosis of hPSCs^17^ and is added to the medium to improve survival following thawing or when iPSCs are passaged as single cells. The medium volume needed will depend on the number of cells and their recovery efficiency. Generally, 10 mL of medium is prepared for immediate thawing before centrifugation, with an additional 4 mL required to fill two 6-wells with 2 mL of medium each. See Figure 2 for details on layout and subsequent medium transfer. We routinely thaw c. 1 × 10^6^ cells/cryovial (clump-based) in this format, with excellent post-thawing recovery and initial passaging within four days of thawing.
  b. A 6-well plate with two wells coated with Laminin Substrate (as described in coating plates with ECMatrix-511 Silk E8 Laminin substrate).
  c. Once ready to use, gently aspirate the coating and add 2 mL of RevitaCell-supplemented StemFlex Medium per coated well.
  d. Place the plate in the humidified incubator to equilibrate to 37°C and the correct pH (5% CO_2_ as required for bicarbonate-buffered media solutions).
    ***Note:*** We recommend warming and pH-equilibrating the medium that will be used for the post-thaw wash of the cells. This can be done by adding a total of 10 mL of RevitaCell-supplemented StemFlex Medium across two of the non-coated 6-wells (see Figure 2) in the original plate to be equilibrated in the incubator.
  e. A cryovial with 1 mL frozen cell suspension, kept on dry ice until ready to process.
    ***Note:*** We do not recommend prolonged (>1 week) storage of iPSCs at −80°C as this will compromise recovery post-thawing. The frozen stocks should be preserved in liquid nitrogen. Cryovials containing cells are stored in the liquid phase of liquid nitrogen; however, storage in the vapour phase is also suitable.
6. Take the cryovial and quickly sterilise with 70% ethanol.
7. Transfer to thaw at 37°C in the humidified incubator.
  a. Leave undisturbed until a tiny ice lump remains (approximately 4 min).

***Note:*** We do not recommend the use of water baths for the thawing of antibiotic-free iPSC cultures. As an alternative to incubator thawing, hold the cryovial tightly in your hands until a small ice lump remains. We have never noticed an adverse effect on cell survival and use this approach with a wide range of cell lines.

8. Bring the cryovial to the biosafety cabinet together with the RevitaCell-supplemented StemFlex Medium.
9. Take up 3 mL of temperature and pH-equilibrated medium.
10. Add 2 mL to the receiving 15 mL Falcon tube and, very slowly, the remaining 1 mL to the cryovial with cells.

**CRITICAL:** This needs to be performed very slowly to avoid osmotic and thermal shock.

11. Immediately, and slowly, transfer the cell suspension to the 15 mL Falcon tube pre-filled with 2 mL of medium.

**CRITICAL:** This needs to be performed slowly and under gentle manual shaking to avoid osmotic and thermal shock.

***Optional:*** Take up another 1 mL of medium from the prefilled wells to wash the cryovial to recover any cells remaining and combine with the cell suspension in the Falcon tube.

12. Take up the remainder of the RevitaCell-supplemented StemFlex Medium from the uncoated 6-wells.
  a. Gently add to the 15 mL Falcon tube with cells.
  b. You should now have 11 mL of cell suspension in total.
13. Return the 6-well plate with the remaining medium in the coated wells to the incubator to keep pH and temperature stable until ready to seed the cells.
14. Centrifuge the cell suspension at 200 *g* for 3 min in a swinging-bucket rotor centrifuge.
15. Remove the supernatant, leaving approximately 50–100 μL of cell suspension behind and taking care not to aspirate the pellet.
16. Flick the tube gently to resuspend the pellet.
17. Take out the 6-well plate and use the medium in the coated well(s) to resuspend the pellet.

**CRITICAL:** Work quickly to avoid drying out of the coated well following aspiration. We recommend taking 1 mL out of the 6-well; this will leave 1 mL in the well, not allowing the coating to dry out while the pellet is being resuspended.

18. Resuspend the pellet gently and dispense the cell suspension into the coated wells.
19. Immediately, move the plate in a “North-South-East-West” direction to distribute the cells.

**CRITICAL:** Take care to achieve a homogenous suspension of smaller cell clumps (>5 cells), as opposed to single cells, to help the iPSCs survive. Avoid larger cell clumps of 20 cells or more, as this will increase the risk of differentiation or poor attachment.

***Note:*** The number of wells to use per thawing may need to be determined empirically based on the initial cell pellet size after centrifugation and knowledge of cell line-specific post-thaw recovery. If unsure, start by seeding into one well, then inspect under the microscope. If the cells are too dense or the clump size too large, take up the medium in the spare well, mix gently into the well containing cells, and dispense 2 mL each across the two wells.

20. Return the cells to the incubator.
  a. Repeat the “North-South-East-West” movement for even dispersion.
21. Monitor cell recovery and remove RevitaCell Supplement after 24 h if colonies have formed successfully and appear to be growing.

***Note:*** Our cryomedium (CTS PSC Cryomedium) does not contain FBS. If your cryomedium contains FBS, exchange the medium 4–8 h after thawing to remove any residual FBS, which may otherwise cause spurious differentiation. The cells should be re-fed with RevitaCell-supplemented medium up to 24 h post-thawing to aid recovery.

### Daily iPSC maintenance

**Timing: 10 min**

**Figure 3:**
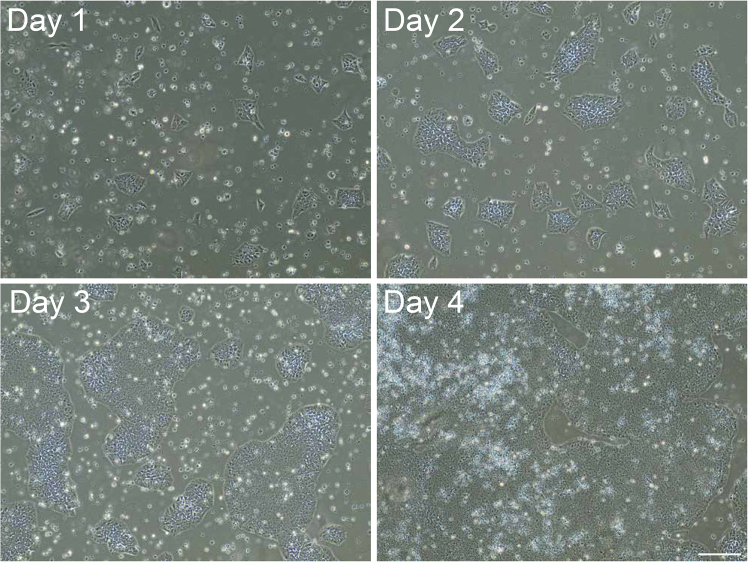
Representative micrographs illustrating healthy iPSC morphology over time Cells were cultured in StemFlex on ECMatrix-511 Silk E8 Laminin Substrate. When colonies begin to merge (Day 4 example), the culture is ready for passaging. Scale bar: 200 μm.

It is important to assess the health of iPSC colonies continually and ensure no spontaneous differentiation is taking place. Healthy iPSCs are small and tightly packed into colonies. Figure 3 provides examples of healthy iPSC colonies to use as a reference. As with all cell culture, the following procedure should be performed in a biosafety cabinet following appropriate aseptic technique.

22. Pre-warm StemFlex Medium to 19°C–23°C before starting.
23. Inspect the cells to assess their health and morphology.
24. Remove old medium from each 6-well containing cells.
25. Gently dispense 2 mL fresh StemFlex Medium along the side of each well, thus avoiding disturbance of the cell monolayer.

***Note:*** We typically achieve 100% undifferentiated iPSC cultures by ensuring consistent daily feeding times with freshly supplemented StemFlex Medium that has been aliquoted in advance. This minimises medium opening/closing, pH buffering loss, and repeated warming of a common stock bottle.

***Note:*** When assessing cell confluency, it is also advisable to examine the edges of the well, as these often provide the most reliable indicator of overall readiness for passaging. Therefore, even if the centre of the well does not appear fully confluent, passaging is recommended if the edges have reached the appropriate confluency threshold.

### Clump-based iPSC passaging with Versene (0.5 mM EDTA)

**Timing: 15 min**

When the iPSCs reach a confluency of approximately 80%–90% (Figure 3; Day 4), they require passaging. We perform clump-based passaging with Versene, which is gentle to the iPSCs, helps them survive, and limits spontaneous differentiation. The following procedure should be performed in a biosafety cabinet following appropriate aseptic technique.

26. Prepare in advance:
  a. A 6-well plate with the required number of wells coated with Laminin Substrate (as described in coating plates with ECMatrix-511 Silk E8 Laminin substrate).
  b. When ready to use, aspirate the coating and add 2 mL of StemFlex Medium per well.
  c. Return the plate to the incubator to allow equilibration to 37°C and the correct pH.
  d. StemFlex Medium and Versene pre-warmed to 19°C–23°C before starting.

***Note:*** We maintain two to three wells per cell line using different densities to achieve at least one optimal cell density for subsequent passaging, depending on the elapsed time (3–5 days).

***Note:*** We recommend passaging iPSCs at least twice after thawing before using them for differentiation experiments.

**Figure 4:**
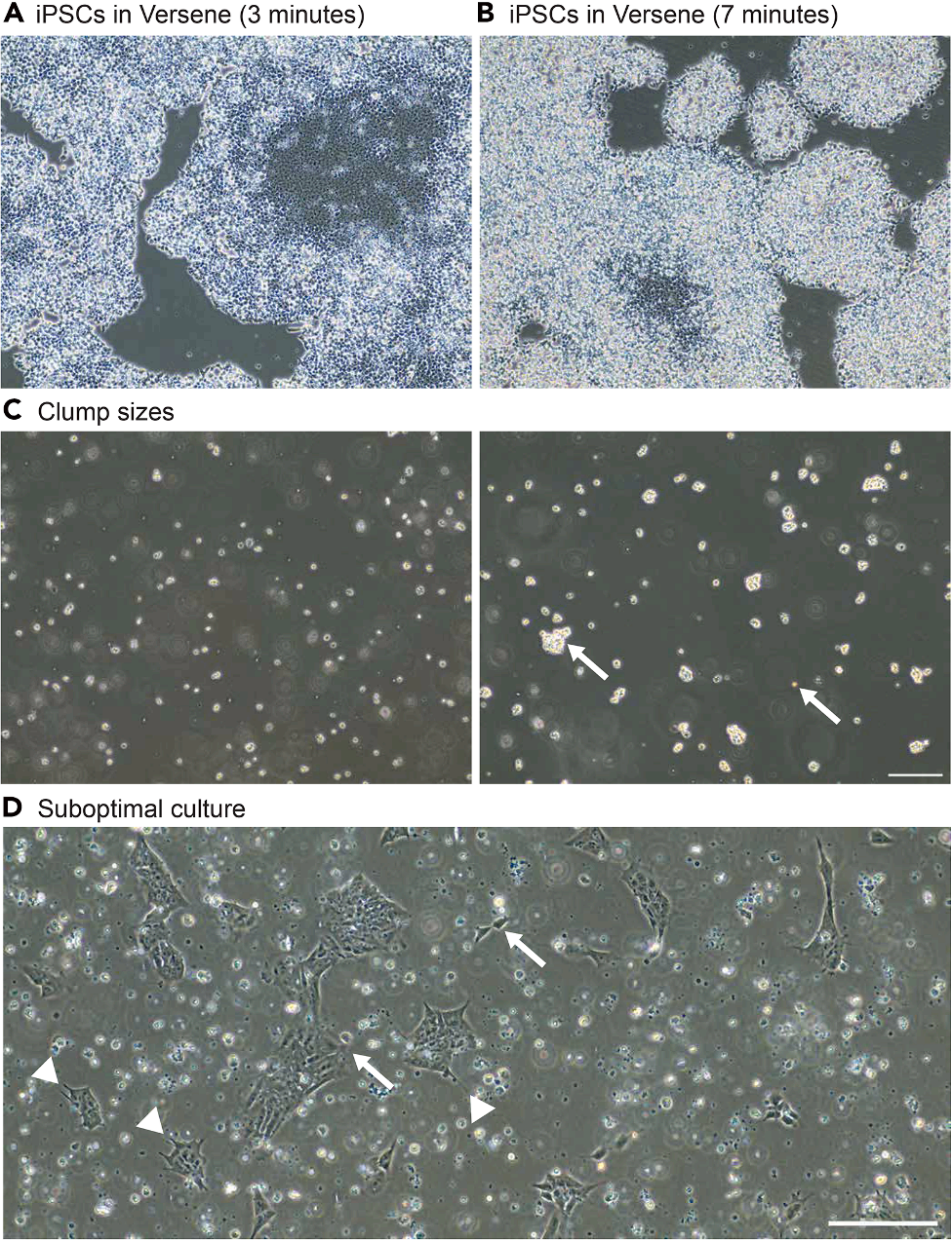
Clump-based iPSC passaging Representative micrographs illustrating cell morphology following non-enzymatic dissociation. (A) Micrograph depicting iPSCs treated with Versene for 3 minutes, which results in insufficient detachment. (B) Micrograph depicting iPSCs treated with Versene for 7 minutes, resulting in optimal detachment prior to processing. (C) Micropgraphs indicating iPSC clump sizes post-dissociation and seeding. White arrows indicate variability in clump size, including the presence of small clumps and occasional single cells. (D) Micrograph depicting iPSCs one day post-split, with suboptimal morphology and variable colony sizes. White arrowheads indicate cellular projections that we use as markers of cell stress. Scale bar: 200 μm. When cultured in StemFlex on ECMatrix-511 Silk E8 laminin substrate, cells exhibit high survival even when dissociated into small clumps of 3-4 cells, without the addition of ROCK inhibitor during the first 24 h. In contrast, this is not observed on Geltrex or Matrigel, and we recommend careful optimisation of passaging conditions to minimise stress following splitting as this can increase spontaneous differentiation.

***Note:*** Check cells under the microscope after the first 6 min to confirm the loosening of colonies. If the core of the colonies appears intact and non-refractile, return the plate to the incubator for another 1–2 min before checking again. Note that the rate of dissociation is slower the larger the colonies are, and will also vary as a function of iPSC line. Some iPSC lines may need longer than 8 min to dissociate fully.

27. Wash the 6-well to be split with 2 mL of DPBS.
28. Treat with 500 μL Versene.
  a. Return to the incubator (37°C) for 6–8 min.
  b. Cells are considered sufficiently detached when they appear as shown in Figure 4B.

**CRITICAL:** Do not leave the iPSCs in Versene too long. The colonies should appear refractile with disrupted cell-cell junctions, but should not be lifting off by themselves. Additionally, in large confluent colonies, the centre may not detach fully (e.g., Figure 4B); if the majority of cells have loosened up, proceed with passaging. The strongly attached cells will remain behind, which helps limit spontaneous differentiation.

29. Return the plate with dissociated colonies to the biosafety cabinet.
  a. Open and tilt the plate at a 45° angle.
  b. Using a P1000 pipette, gently take up the Versene solution with cells,
  c. Dispense back across the well in a systematic motion.

**CRITICAL:** Do not scratch the surface of the plate. Instead, pipette across the well gently, just above the cells, and avoid excessive stress. Move systematically from one side to the other, from the top to the bottom of the well, whilst taking care not to pipette over the same area excessively. After doing this 4–5 times, you should observe that most cells have dissociated from the well.

30. Transfer the entire solution into a sterile 1.5 mL Eppendorf.
31. Split according to your desired ratio by taking the required volume from the dissociated cell suspension into the well pre-filled with 2 mL of StemFlex Medium.

***Note:*** For example, for a 1:20 split, you would take 25 μl of dissociated cells (out of 500 μL total volume). The usual ratios we use range between 1:5 and 1:20 without RevitaCell/ROCK inhibitor supplementation when cells are cultured on the recommended laminin substrate. Optimal ratios need to be determined empirically for each iPSC line. For the WTC11 iPSC line, an optimal split ratio of 1:15-1:20 will result in iPSC colonies that are ready for split 4–5 days later.

32. Immediately after dispensing the cells, gently move the plate according to a “North-South-East-West” movement for an even distribution.
33. Briefly inspect the cells under the microscope to confirm the correct clump size has been achieved (see example in Figure 4C).

***Note:*** If cell clumps are more heterogenous than desired, e.g., as shown in Figure 4C, there is an increased risk of uneven attachment (Figure 4D) and unwanted differentiation. We recommend taking the plate back into the biosafety cabinet and gently pipetting up and down in the well with a P1000 pipette to break up larger clumps. Do not pipette more than 5 times to minimise excessive stress on the cells, and take care not to disturb the laminin coating.

34. Return the plate with cells back to the incubator.
  a. Repeat the “North-South-East-West” movement for even dispersion.

### Differentiation media preparation

**Timing: 24 h (2 h hands-on)**

**Figure 5:**
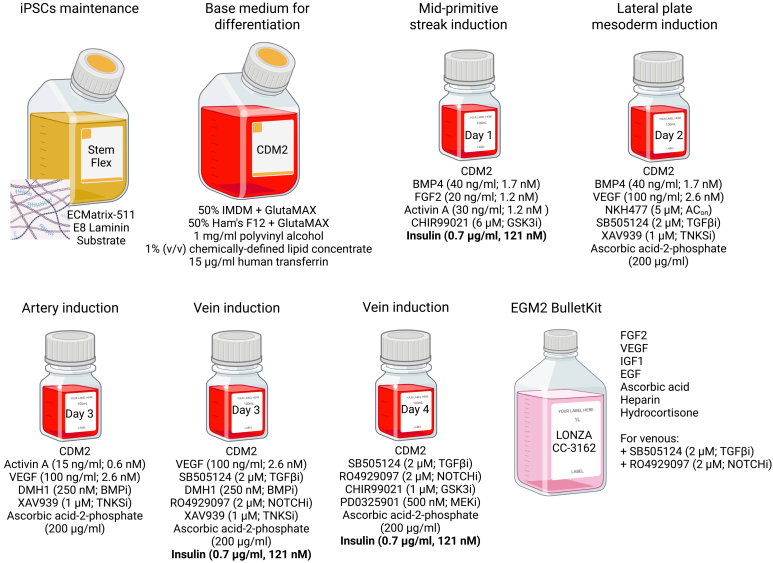
Schematic illustrating all media solutions required for the protocol Molar concentrations are approximate, as some growth factor preparations may contain mixtures of different isoforms, giving rise to a mixture of molecular weights. Note that the CDM2 base medium is free of any PI3K-activating growth factors. Created in BioRender. Madsen, R. (2026) https://BioRender.com/eb8fnnf.

Acquire all media and reagents as specified in *Materials and Equipment* (see also Figure 5 for an overview). The following procedures should be performed in a biosafety cabinet following appropriate aseptic technique, and reagents should be sterile-filtered where necessary. Refer to Supplementary File 1 for a pre-formatted Excel template with embedded formulas for media-specific component concentrations and volume calculations.

35. Reconstitute all commercial reagents following the manufacturers’ instructions.
36. Prepare 10 mL of 50 mg/mL PVA solution:
  a. Weigh 500 mg of PVA powder and transfer to a 50 mL glass bottle.
  b. Spread the powder evenly across the bottle surface to avoid clump formation.
  c. Place a magnet inside the bottle and transfer to a magnetic, hot plate stirrer.
  d. Add 10 mL of UltraPure DNase/RNase-Free Distilled Water drop-wise while stirring.
  e. Close the bottle and heat to 85°C while mixing until the powder dissolves (1–2 h).
  f. Cool the solution to 19°C–23°C while stirring for 16–20 h.
  g. Transfer the bottle to biosafety cabinet and sterile-filter (0.22 μm PES) into a clean container.
  h. Store at 4°C for up to 6 months.

**Alternatives**: If a hot plate stirrer is not available, a water bath set to 85°C may be used instead. In this case, mix intermittently during heating until fully dissolved, then allow the solution to cool to 19°C–23°C for 16–20 h on a magnetic plate stirrer.

37. Prepare CDM2 Base Medium by combining all reagents as specified under final concentration in *Materials and Equipment.*
38. Sterile-filter the CDM2 solution through a 0.22 μm PES membrane.

***Note:*** The CDM2 Base Medium can be stored in the fridge (4°C) for up to 1 month, provided that repeated opening/closing is kept to a minimum, or the medium subaliquoted, to preserve the 1-thioglycerol.

39. Prepare all stage-specific differentiation media solutions as specified under final concentration *Materials and Equipment.*

***Note:*** Store the stage-specific differentiation media solutions in the fridge (4°C) and use within a week of preparation. While differentiation medium may be stored for up to 1 week, optimal performance is achieved when the medium is supplemented fresh or shortly before use to limit degradation of sensitive components. Heating the medium is not recommended. Aliquots should be equilibrated to 19°C–23°C before use.

40. Prepare the EGM-2 Complete Medium as specified according to the manufacturer’s instructions.

**CRITICAL:** Arterial-like endothelial cells (ALECs) are cultured in EGM-2 Complete Medium. Venous-like endothelial cells (VLECs) require supplementation of EGM-2 Complete Medium with RO4929097 and SB505124, both at a final concentration of 2 μM, as described previously.^10^

***Note:*** We omit the addition of GA-1000 (Gentamicin and Amphotericin) from the EBM-2 Endothelial Cell Growth Medium-2 for antibiotic-free culture.

***Note:*** EGM-2 Complete Medium may be prepared in advance and stored at 4°C for up to 1 month according to the manufacturer’s recommendations. However, because human fibroblast growth factor basic (bFGF or FGF2) is relatively unstable, we recommend aliquoting the basal medium and supplements separately, with supplements stored at −80°C. Complete medium should then be prepared as required and stored for up to 2 weeks after preparation to minimize the risk of reagent activity loss at 4°C. Similar precautions apply to StemFlex Medium preparation.

## Key resources table

**Table:** 

REAGENT or RESOURCE	SOURCE	IDENTIFIER
**Antibodies**		

anti-Oct3/4 (clone C-10) – mouse monoclonal, 1:100	Santa Cruz Biotechnology	Cat# sc-5279
anti-Nanog – rabbit polyclonal, 1:200	Thermo Fisher Scientific	Cat# PA1-097
anti-CD144 (VE-cadherin) (clone 55-7H1) – mouse monoclonal, 1:200	BD Pharmingen	Cat# 555661
anti-SOX17 – goat polyclonal, 1:200	Bio-Techne	Cat# AF1924
anti-NR2F2 (clone EPR18443) conjugated to Alexa Fluor 594 – rabbit monoclonal, 1:100	Abcam	Cat# ab311744
anti-NR2F2 (clone EPR18443) – rabbit monoclonal, 1:100	Abcam	Cat# ab211777
anti-Ki-67 (clone D3B5) conjugated to Alexa Fluor 488 – rabbit monoclonal, 1:100	CST	Cat# 11882
α-smooth muscle actin (SMA) (clone 1A4) – mouse monoclonal, 1:500	Thermo Fisher Scientific	Cat# 14-9760-82
anti-goat highly cross-adsorbed secondary antibody conjugated to Alexa Fluor 488 – donkey polyclonal, 1:1000	Thermo Fisher Scientific	Cat# A32814
anti-rabbit highly cross-adsorbed secondary antibody conjugated to Alexa Fluor 555 – donkey polyclonal, 1:2000	Thermo Fisher Scientific	Cat# A31572
anti-rabbit highly cross-adsorbed secondary antibody conjugated to Alexa Fluor 594 – donkey polyclonal, 1:2000	Thermo Fisher Scientific	Cat# A32754
anti-rabbit highly cross-adsorbed secondary antibody conjugated to Alexa Fluor 594 – goat polyclonal, 1:2000	Thermo Fisher Scientific	Cat# A11037
anti-mouse highly cross-adsorbed secondary antibody conjugated to Alexa Fluor 647 – donkey polyclonal, 1:1000	Thermo Fisher Scientific	Cat# A32787

**Chemicals, peptides, and recombinant proteins**		

StemFlex Medium Kit	Thermo Fisher Scientific	Cat# A3349401
ECMatrix-511 Silk E8 Laminin Substrate	Sigma-Aldrich	Cat# CC161-1050UG
Versene	Thermo Fisher Scientific	Cat# 15040066
StemPro Accutase	Thermo Fisher Scientific	Cat# A1110501
Dulbecco’s phosphate-buffered saline (DPBS)	Thermo Fisher Scientific	Cat# 14190
CTS PSC Cryomedium	Thermo Fisher Scientific	Cat# A4238801
RevitaCell Supplement (10X)	Thermo Fisher Scientific	Cat# A2644501
IMDM (1X) + GlutaMAX-I	Thermo Fisher Scientific	Cat# 31980030
F12 (1X) + GlutaMAX-I	Thermo Fisher Scientific	Cat# 31765035
Polyvinyl alcohol (PVA, 87%–90% hydrolyzed, average molecular weight 30,000–70,000)	Sigma-Aldrich	Cat# P8136
Chemically defined lipid concentrate	Thermo Fisher Scientific	Cat# 11905031
Human transferrin	Sigma-Aldrich	Cat# T8158
1-thioglycerol	Sigma-Aldrich	Cat# M6145
Human recombinant insulin	Sigma-Aldrich	Cat# I9278
Human recombinant BMP4	Thermo Fisher Scientific	Cat# 120-05
Human recombinant FGF-2	Thermo Fisher Scientific	Cat# 100-18B
Human/mouse/rat recombinant Activin A	Thermo Fisher Scientific	Cat# 120-14P
CHIR99021	Bio-Techne	Cat# 4423
Human recombinant VEGF-165	Thermo Fisher Scientific	Cat# 100-20
NKH 477	Sigma-Aldrich	Cat# N3290
SB505124	SelleckChem	Cat# S2186
XAV939	SelleckChem	Cat# S1180
Ascorbic acid-2-phosphate	Sigma-Aldrich	Cat# A8960
DMH1	Sigma-Aldrich	Cat# 203646
RO4929097	SelleckChem	Cat# S1575
PD0325901	SelleckChem	Cat# S1036
EGM-2 Endothelial Cell Growth Medium-2 BulletKit	Lonza	Cat# CC-3162
Bovine Serum Albumin (BSA) 10% in DPBS	Sigma-Aldrich	Cat# A1595
Ethylenediaminetetraacetic (EDTA) acid disodium salt solution	Sigma-Aldrich	Cat# 03690
UltraPure 0.5M EDTA, pH 8.0	Thermo Fisher Scientific	Cat# 15575-038
CD144 (VE-cadherin) MicroBeads, human	Miltenyi Biotec	Cat# 130-097-857
Hoechst 33342	Sigma-Aldrich	Cat# 14533
10X PBS (for immunofluorescence)	Severn Biotech	Cat# 20-7400
Tween-20	Promega	Cat# H5152
Triton X-100	Promega	Cat# H5141
10X fish skin gelatin blocking agent (FSG)	Biotium	Cat# 22010
UltraPure DNase/RNase-Free Distilled Water	Thermo Fisher Scientific	Cat# 10977035

**Experimental models: Cell lines**		

WTC11 iPSC line (Japanese, male, from fibroblast of skin, 30-years-old)	Coriell Institute	Cat# GM25256 RRID: CVCL_Y803
Mono-allelic mEGFP-tagged CDH5 WTC iPSC line (CRISPR-derivative of WTC11)	Coriell Institute	Cat# AICS-0126-068

**Other**		

Nunc Cell-Culture Treated Multidishes – 6-well	Thermo Fisher Scientific	Cat# 140685
Nunc Cell-Culture Treated Multidishes – 12-well	Thermo Fisher Scientific	Cat# 150628
ViewPlate-96 TC black	PerkinElmer	Cat# 6005182
PhenoPlate-96 TC black	Revvity	Cat# 6055300
MycoAlert mycoplasma detection kit	Lonza	Cat# LT07-318
Venor GeM qOneStep qPCR mycoplasma detection kit	Minerva Biolabs	Cat# 11-91025
Millipore Stericup Quick Release Vacuum Filtration System, 0.22 μm, PES	Sigma-Aldrich	Cat# S2GPU05RE
Nalgene Rapid-Flow Sterile Disposable Filter Unit, 0.2 μm, PES	Thermo Fisher Scientific	Cat# 566-0020
pluriStrainer Mini cell strainer, mesh size 40 μm	pluriSelect	Cat# 43-10040-50
QuadroMACS Separator	Miltenyi Biotec	Cat# 130-090-976
MACS MultiStand	Miltenyi Biotec	Cat# 130-042-303
LS Columns	Miltenyi Biotec	Cat# 130-042-401
Corning Elplasia 96-well round bottom microplate, ULA surface	Corning	Cat# 4442
25 mL, sterile reservoir	Integra	Cat# 4312
300 μL 8 Channel VIAFLO electronic multichannel pipette	Integra	Cat# 4623
RoboRocker 212	NextAdvance	Cat# RR212-PL.G
405 Select TS Washer with 4 buffer bottles	Agilent	Cat# 405TSUV-SN
Vacuum system with high flow for plate washer	Agilent	Cat# 7100753S

***Note:*** We recommend tracking of LOT numbers and expiry dates for each of the components. It is critical to use high-quality reagents that have not expired as iPSCs are very sensitive to suboptimal culture conditions.

***Note:*** We recommend switching the light off in the biosafety cabinet to protect light-sensitive reagents when specified by the manufacturer.

***Note:*** Due to the hygroscopic nature of DMSO, care should be taken to minimize water absorption. Stocks should be handled quickly and all tubes securely sealed (e.g., with Parafilm) to prevent dilution.

## Materials and equipment

**Table:** CDM2 Base Medium stock and final concentrations of reagents

Reagent	Solvent	Stock concentration	Final concentration
IMDM (1X) + GlutaMAX-I	N/A	100%	50%
F12 (1X) + GlutaMAX-I	N/A	100%	50%
Polyvinyl alcohol (PVA)	water	50 mg/mL	1 mg/mL
Chemically defined lipid concentrate	N/A	100%	1% (v/v)
Human transferrin	water	30 mg/mL	15 μg/mL
1-thioglycerol	N/A	11.5 M	450 μM

Store for up to 1 month at 4°C.

**Table:** Stage-specific differentiation media solutions and final concentrations of reagents

Reagent	Solvent	Stock concentration	Final concentration
**Day 1: Mid primitive streak induction**			
CDM2 base medium	N/A	N/A	N/A
Human recombinant insulin	HEPES	10 mg/mL	0.7 μg/mL
Human recombinant BMP4	5 mM HCl	20 μg/mL	40 ng/mL
Human recombinant FGF-2	5 mM Tris-HCl	30 μg/mL	20 ng/mL
Human/mouse/rat recombinant Activin A	water	100 μg/mL	30 ng/mL
CHIR99021	DMSO	10 mM	6 μM
**Day 2: Lateral plate mesoderm induction**			
CDM2 base medium	N/A	N/A	N/A
Human recombinant BMP4	5 mM HCl	20 μg/mL	40 ng/mL
Human recombinant VEGF-165	water	100 μg/mL	100 ng/mL
NKH 477	water	10 mM	5 μM
SB505124	DMSO	10 mM	2 μM
XAV939	DMSO	10 mM	1 μM
Ascorbic acid-2-phosphate	water	50 mg/mL	200 μg/mL
**Day 3: ALEC induction**			
CDM2 base medium	N/A	N/A	N/A
Human recombinant VEGF-165	water	100 μg/mL	100 ng/mL
Human/mouse/rat recombinant Activin A	water	100 μg/mL	15 ng/mL
DMH1	DMSO	1 mM	250 nM
XAV939	DMSO	10 mM	1 μM
Ascorbic acid-2-phosphate	water	50 mg/mL	200 μg/mL
**Day 3: VLEC induction day 1**			
CDM2 base medium	N/A	N/A	N/A
Human recombinant insulin	HEPES	10 mg/mL	0.7 μg/mL
Human recombinant VEGF-165	water	100 μg/mL	100 ng/mL
SB505124	DMSO	10 mM	2 μM
DMH1	DMSO	1 mM	250 nM
RO4929097	DMSO	10 mM	2 μM
XAV939	DMSO	10 mM	1 μM
Ascorbic acid-2-phosphate	water	50 mg/mL	200 μg/mL
**Day 4: VLEC induction day 2**			
CDM2 base medium	N/A	N/A	N/A
Human recombinant insulin	HEPES	10 mg/mL	0.7 μg/mL
SB505124	DMSO	10 mM	2 μM
RO4929097	DMSO	10 mM	2 μM
CHIR99021	DMSO	10 mM	1 μM
PD0325901	DMSO	1 mM	500 nM
Ascorbic acid-2-phosphate	water	50 mg/mL	200 μg/mL

Store for up to 1 week at 4°C.

**Table:** MACS buffer stock and final concentrations of reagents

Reagent	Solvent	Stock concentration	Final concentration
DPBS	N/A	N/A	N/A
Bovine serum albumin (BSA)	DPBS	10%	0.5%
EDTA	water	500 mM	2 mM

Store for up to 1 year at 4°C.

## Step-by-step method details

**Figure 6:**
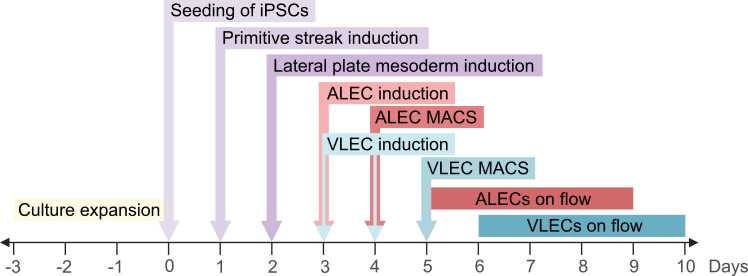
2D differentiation timeline overview ALEC, arterial-like endothelial cell; VLEC, venous-like endothelial cell.

This workflow outlines the generation of ALECs and VLECs from iPSCs in a 2D monolayer format. An alternative 3D format is also presented (*Step 85* onwards). Step-by-step instructions are provided for initial seeding, directed differentiation, purification using magnetic-activated cell sorting (MACS), and subsequent maturation of endothelial cells on flow (Figure 6). The following procedures should be performed in a biosafety cabinet following appropriate aseptic technique.

**CRITICAL:** No antibiotics are added to either iPSC or differentiating culture media. All steps must be performed under sterile conditions. If the medium appears cloudy or cells show unexpected signs of death, discard the culture and start with a fresh cell suspension. We also recommend weekly testing for mycoplasma using either PCR-based or luminescence workflows as per manufacturers’ instructions (see also key resources table).

### Experimental seeding of iPSCs

**Timing: 1 h**

This section outlines the initial seeding of iPSCs at optimal densities for growth and differentiation. The iPSCs should be harvested when subconfluent, as we have seen better differentiation results with cells that were around 70%–80% dense.

1. Prepare in advance:
  a. The required volume of StemFlex Medium supplemented with RevitaCell Supplement diluted to 1X, pre-warmed to 19°C–23°C.
    ***Note:*** Each 6-well with iPSCs to be used for differentiation will be passaged with 500 μL of StemPro Accutase; to facilitate enzymatic inactivation, we collect the dissociated cells in StemFlex Medium with RevitaCell corresponding to 3 times the volume of Accutase. We therefore recommend aliquoting this collecting medium in advance. For example, if three iPSC 6-wells are to be split, prepare a tube containing 4.5 mL of medium. Additionally, each recipient well for differentiation should will require 2 mL of medium for seeding.
  b. A tissue culture-treated 6-well plate with the desired number of coated wells to receive cells (as described in coating plates with ECMatrix-511 Silk E8 Laminin substrate).
  c. Once the plates have been incubated for a sufficient period, gently aspirate the coating.
  d. Immediately, add 1.5 mL of StemFlex Medium supplemented with RevitaCell per coated well.
  e. Put the plate in the incubator to equilibrate to 37°C and the correct pH.
    ***Note:*** The number of wells to be seeded depends on the required cell numbers for downstream applications. At a minimum, we recommend seeding four to six 6-well plates. Although yields may vary, seeding from six 6-well plates typically allows for the generation of approximately six to twelve 12-wells for ALECs and four to eight 12-wells for VLECs.
  f. A 1.5 mL Eppendorf pre-filled with 20 μL of 0.4% Trypan Blue for counting.
  g. StemPro Accutase pre-warmed to 19°C–23°C before starting.
    ***Note:*** We use StemPro Accutase for gentle single-cell-based passaging to allow for counting of cells for precise seeding.
2. Wash each 6-well to be split with 2 mL of DPBS.

***Note:*** For seeding of six 6-wells (one full 6-well plate) for differentiation, start with three subconfluent 6-wells of iPSCs.

3. Treat each 6-well with 500 μL of StemPro Accutase and return to the incubator (37°C) for 6–8 min.

***Note:*** Check cells under the microscope after the first 6 min to confirm the loosening of colonies. If not ready, put back in the incubator for another minute before checking again. The detachment speed will be iPSC line-specific.

4. Return the plate with dissociated colonies to the biosafety cabinet.
  a. Open and tilt the plate at a 45° angle.
  b. Using a P1000 pipette, gently take up the StemPro Accutase solution with cells.
  c. Dispense back across the well in a systematic motion.

**CRITICAL:** Do not scratch the surface of the plate. Instead, pipette across the well gently, just above the cells, and avoid excessive stress. Move systematically from one side to the other, from the top to the bottom of the well, whilst taking care not to pipette over the same area excessively. After doing this 4–5 times, you should observe that most cells have dissociated from the well.

5. Transfer the entire solution into a tube filled with a sufficient amount of collecting medium, as outlined in *Step 1a* and the associated *Note.*
6. Take 20 μL of the cell suspension for counting.
  a. Add it to the Eppendorf tube containing Trypan Blue, as outlined in *Step 1f.*
  b. Mix thoroughly, then load 10 μL into a haemocytometer and count the cells manually.

***Note:*** We recommend manual counting, as it gives more consistent seeding results compared with automated counters.

7. Calculate the total volume of cells required for seeding each differentiation condition.

**Table 1:** Recommended cell numbers for differentiation initiation of arterial-like (ALECs) and venous-like endothelial cells (VLECs)

Differentiation condition	Target cell seeding density (cells/cm^2^)	Target cells per 6-well	Cells per well with 10% overage	Cell suspension concentration (cells/mL)
ALECs	45,000	432,000	475,200	950,400
VLECs	30,000	288,000	316,800	633,600

***Note:*** For arterial differentiation, seed 45,000 cells/cm^2^; for venous differentiation, 30,000 cells/cm^2^. Refer to Table 1 for the total number of cells required per well. We add an additional 10% for the cell numbers to account for loss during centrifugation. We also calculate for seeding of an extra well, i.e., seven if we aim to seed six final, to minimise the effect of pipetting loss following resuspension. For example, to seed cells into six wells in a 6-well plate format for ALEC differentiation, we take forward a total of 3.33 × 10^6^ cells for seven wells total, including the 10% overage.

8. Transfer the required volume of cells into a new 15 mL Falcon.
9. Centrifuge at 200 *g* for 3 min in a swinging-bucket rotor centrifuge.
10. Remove the supernatant, leaving approximately 50–100 μL of cell suspension behind and taking care not to disturb the pellet.
11. Flick the tube gently to resuspend the pellet.
12. Resuspend in the required volume of StemFlex Medium supplemented with RevitaCell.

***Note:*** For example, for seeding of six 6-wells, add a total of 3.5 mL (i.e., you will be seeding 500 μL into each well pre-filled with 1.5 mL medium, and as you have calculated for a total of seven wells, there should be about 500 μl left once done).

13. Mix gently to fully resuspend the cells in the medium.
14. Bring the recipient pre-filled and coated plate (see *Steps 1b-c*) to the biosafety cabinet.
15. Dispense 500 μL of cell suspension per well.
16. Immediately after dispensing the cells, move the plate in a “North-South-East-West” direction to distribute the cells.
17. Return the cells to the incubator and repeat the “North-South-East-West” movement for even dispersion.

### Lineage-specific induction

**Timing: 3–4 days (30 min per day)**

**Figure 7:**
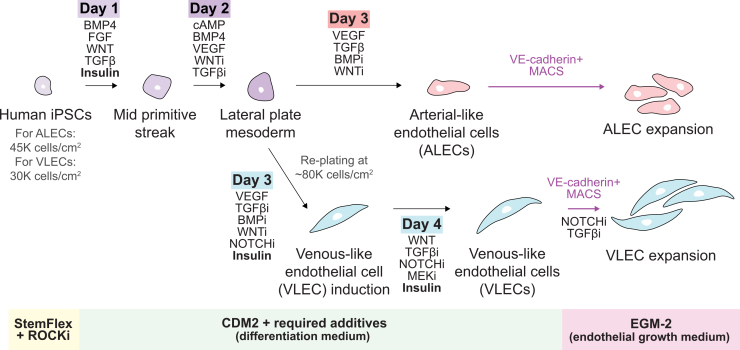
Overview of lineage-specific induction steps Note the absence of insulin (physiological class IA PI3K activator) and GDC0941 (pharmacological class I PI3K inhibitor when used at 2.5 μM) at the lateral plate mesoderm and ALEC induction steps, a key difference from the original protocol.^10^ K denotes ×1,000.

**Figure 8:**
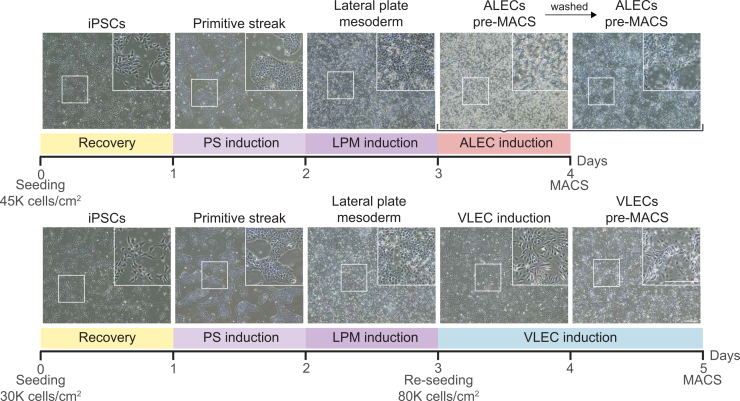
Expected cell densities and morphologies of the cells at each differentiation stage Scale bar: 200 μm. K denotes ×1,000.

This section outlines the steps for the directed differentiation from iPSCs into arterial-like (ALECs) and venous-like endothelial cells (VLECs) (Figure 7) using the CDM2 Base Medium with stage-specific supplementations (see also differentiation media preparation and Figure 5). For reference, expected densities and morphologies of differentiating cells can be seen in Figure 8.

18. In advance, pre-warm the required volume of the relevant differentiation medium and DMEM/F-12 (no glutamine) to 19°C–23°C.

**CRITICAL:** Aliquot the media to maximise shelf life and maintain buffering capacity for consistent performance.

**CRITICAL:** Individual medium changes at each differentiation should occur at consistent 24 h intervals, with a maximum variation of ± 60 min allowed.

### Arterial lineage differentiation

19. Remove the previous day’s medium from each well.
20. Wash with 4 mL of DMEM/F-12 (no glutamine) per well.

**CRITICAL:** When washing the cells, dispense 1 mL of medium first to each well and top up with the remaining 3 mL only once all wells contain medium to avoid wells drying out and to minimise inconsistent wash timings across wells. Ensure you dispense the medium on the side of the well, quickly but gently, to avoid perturbing the cells. Cells should not be left in the wash medium for extended periods as this can impact the downstream differentiation negatively.

21. Add 2 mL of appropriate differentiation medium per well.

***Note:*** One day after arterial differentiation induction, substantial cell death is typically observed, along with the emergence of tube-like structures connecting individual cell clusters (see Figure 8 for ALECs pre-MACS before and after a double wash with DPBS to remove debris).

### Venous lineage differentiation

22. For Day 1 (mid primitive streak) and Day 2 (lateral plate mesoderm) inductions: remove the previous day’s medium from each well.
  a. Wash with 4 mL of DMEM/F-12 (no glutamine) per well as described in *Step 20* and the associated *Critical Note.*
23. Add 2 mL of the appropriate differentiation medium per well.

**CRITICAL:** For both ALEC and VLEC differentiation, these steps should be repeated every 24 h, with a maximum variation of ± 60 min allowed.

Following differentiation into lateral plate mesoderm, the cells require re-seeding to adjust for cell density and improve the venous differentiation efficiency in 2D.

24. Prepare in advance:
  a. The required volume of Day 3 (VLEC induction day 1) medium, pre-warmed to 19–23°C.
    ***Note:*** Each 6-well will be passaged with 500 μL of StemPro Accutase. This must be inactivated by adding 3 times the volume of Day 3 medium to stop enzymatic activity. We therefore recommend aliquoting this collecting medium in advance. For example, if six wells are to be split, prepare a tube containing 9 mL of collecting medium. Additionally, each recipient well will require 2 mL of medium for seeding.
  b. A 6-well plate with the desired number of coated wells (as described in coating plates with ECMatrix-511 Silk E8 Laminin substrate). Gently aspirate the coating and add 1 mL of Day 3 (VLEC induction day 1) differentiation medium per coated well. Transfer the plate to the incubator to equilibrate to 37°C and the correct pH.
    ***Note:*** The number of wells to be seeded into will depend on the number of wells available for passaging and their yield following dissociation. We are typically able to re-seed cells into nine 6-wells when we collect cells from a full 6-well plate.
  c. A 1.5 mL Eppendorf pre-filled with 20 μL of 0.4% Trypan Blue for counting.
25. Pre-warm StemPro Accutase to 19°C–23°C before starting.
26. Wash each 6-well to be split with 2 mL of DPBS.
27. Treat each well with 500 μL of StemPro Accutase.
  a. Return to the incubator (37°C) for 5 min.

***Note:*** Check under the microscope after the first 3–4 min to confirm the loosening of cells. The detachment of these cells is much faster than iPSCs, so prepare to process them quickly.

28. Return the plate with dissociated cells to the biosafety cabinet.
  a. Open and tilt the plate at a 45° angle.
  b. Using a P1000 pipette, gently take up the StemPro Accutase solution with cells.
  c. Dispense back across the well in a systematic motion.

**CRITICAL:** Do not scratch the surface of the plate. Instead, pipette across the well gently, just above the cells, and avoid excessive stress. Move systematically from one side to the other, from the top to the bottom of the well, whilst taking care not to pipette over the same area excessively. After doing this 4–5 times, you should observe that most cells have dissociated from the well.

29. Transfer the entire solution into a tube filled with a sufficient amount of collecting medium, as outlined in *Step 24a.*
30. Take 20 μL of the cell suspension for counting.
  a. Add to the Eppendorf tube containing Trypan Blue, as outlined in *Step 24c.*
  b. Mix thoroughly, then load 10 μL into a haemocytometer and count the cells manually.
31. Calculate the volume of cells required for 80,000 cells/cm^2^ re-seeding.
  a. Refer to Table 2, including the 10% overage and volume for an extra well for seeding as described in *Step 7.*

**Table 2:** Recommended cell numbers for re-seeding of differentiating venous-like endothelial cells (VLECs)

Differentiation condition	Target cell seeding density (cells/cm^2^)	Target cells per 6-well	Cells per well with 10% overage	Cell suspension concentration (cells/mL)
VLECs re-seeding	80,000	768,000	844,800	844,800
32. Take up the required volume of cells and transfer to a new tube.

***Note:*** For example, to seed nine 6-wells, collect 8.03 × 10^6^ cells for nine and a half wells total (including the excess).

33. Centrifuge the required volume of cell suspension at 200 *g* for 3 min in a swinging-bucket rotor centrifuge.
34. Remove the supernatant, leaving approximately 50–100 μL of cell suspension behind and taking care not to disturb the pellet.
35. Flick the tube gently to resuspend the pellet.
36. Add the required volume of Day 3 (VLEC induction day 1) medium.

***Note:*** For example, for seeding of nine 6-wells (nine and a half wells with extra), add a total of 9.5 mL.

37. Mix gently to fully resuspend the cells in the medium.
38. Bring the recipient pre-filled plate with coated wells and pre-dispensed medium (see *Step 24b*) to the biosafety cabinet.
39. Dispense 1 mL of well-mixed cell suspension per well.
40. Immediately after dispensing the cells, move the plate in a “North-South-East-West” direction to distribute the cells.
41. Return the cells to the incubator.
  a. Repeat the “North-South-East-West” movement for even dispersion.
42. Following 24 h of incubation, remove the previous day’s medium from each well.
43. Wash with 4 mL of DMEM/F-12 (no glutamine) as described previously in *Lineage Specific Induction Step 20.*
44. Add 2 mL of Day 4 (VLEC induction day 2) medium per well.

### Magnetic-activated cell sorting for CD144 (VE-cadherin) to enrich endothelial cell populations

**Timing: 2–4 h**

This section provides step-by-step instructions for enrichment of pure endothelial cell populations using magnetic microbeads conjugated to an anti-VE-cadherin antibody.

**Figure 9:**
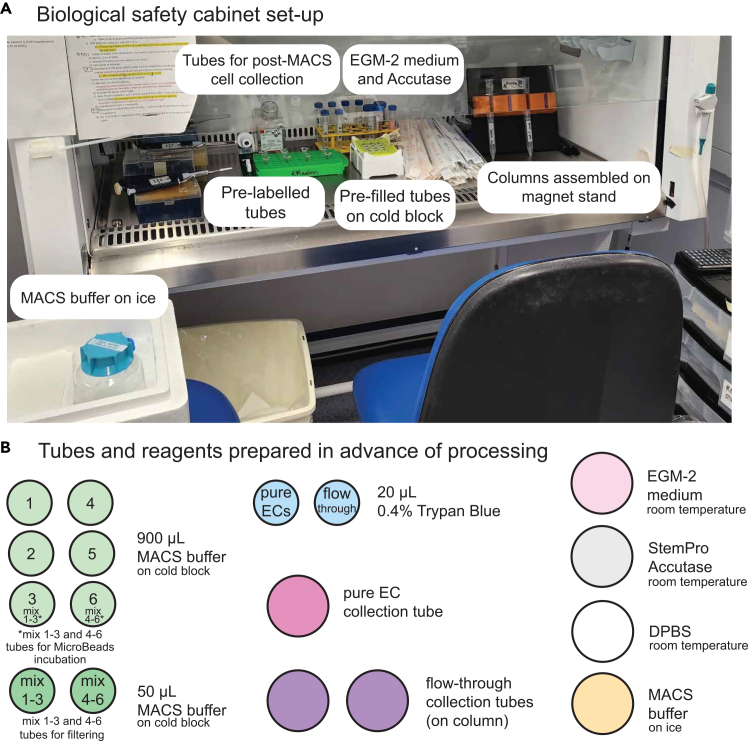
Set-up for MACS in a biological safety cabinet (A) Photograph of a biological safety cabinet with all required materials and reagents for execution of the magnetic-activated cell sorting (MACS) workflow. (B) Graphical overview of all the tubes and key reagents needed for MACS. The set-up shown here is for processing of the same culture seeded across a full 6-well plate. Smaller-sized circles represent 1.5 mL Eppendorf tubes, and larger-sized circles represent larger collection tubes, such as 15 mL or 50 mL Falcon tubes (depending on volumes needed). EC, endothelial cell; MACS, magnetic-activated cell sorting.

45. Prepare in advance:
  a. MACS buffer, kept cold in the fridge (4°C) or on ice during the duration of the procedure (see materials and equipment for recipe).
  b. Add 900 μL of cold MACS buffer to a 1.5 mL Eppendorf tube for each well that will be processed.
    i. For example, if processing 6 wells per cell line, then prepare 6 Eppendorf tubes.
    ii. Keep the tubes on a cold block.
  c. Additional 1.5 mL Eppendorf tubes, pre-filled with 50 μL of cold MACS buffer and labelled with the sample name.
    i. Keep the tubes on a cold block.
      ***Note:*** The number of tubes will depend on the number of processed wells. We have found that, for the best results, cells from a maximum of three 6-wells should be used per MACS column. For example, if processing six 6-wells per sample, prepare two tubes with 50 μl MACS buffer each.
  d. Additional 1.5 mL Eppendorf tube(s) pre-filled with 20 μL of 0.4% Trypan Blue for counting.
    ***Optional:*** Only one tube per clone is required; however, we also recommend preparing an additional tube per clone to count flow-through cells for calculations of differentiation efficiency.
  e. EGM-2 Complete Medium, pre-warmed to 19°C–23°C, to receive ALECs and VLECs post-MACS.
    **CRITICAL:** As outlined in differentiation media preparation*, Step 40 and the associated Critical note*, the EGM-2 Complete Medium for culture of VLECs requires the addition of 2 μM RO4929097 and 2 μM SB505124.
  f. StemPro Accutase, pre-warmed to 19°C–23°C.
  g. A laminin-coated tissue culture-treated 12-well plate(s) (please refer to coating plates with ECMatrix-511 Silk E8 Laminin substrate) to receive cells post-MACS.
    ***Note:*** The number of wells to be seeded into will depend on the efficiency of the differentiation process, the starting number of cells, and the amount of cell loss during MACS. For ALECs, we recommend coating up to twelve wells in a 12-well plate if processing a total of six 6-wells for MACS. For VLECs, we recommend coating up to eight wells in a 12-well plate if processing a total of nine 6-wells for MACS post-reseeding. This may vary if using a different cell line and should be determined empirically.
  h. All else required inside the biosafety cabinet for MACS, including specific materials, beads, and magnets.
  i. Please refer to Figure 9 and *Materials and equipment* for further guidance on set-up and media preparations, respectively.
46. Wash each 6-well to be dissociated with 2 mL of DPBS.
47. Repeat the wash.

**CRITICAL:** Due to more extensive cell death at the last step of arterial differentiation, it is important to wash the cells with DPBS twice.

48. Treat the cells with 500 μL StemPro Accutase and return to the incubator (37°C) for 5 min.
49. Return the plate with dissociated colonies to the biosafety cabinet.
  a. Open and tilt the plate at a 45° angle.
  b. Using a P1000 pipette, gently take up the StemPro Accutase solution with cells.
  c. Dispense back across the well in a systematic motion.

**CRITICAL:** Do not scratch the surface of the plate. Instead, pipette across the well gently, just above the cells, and avoid excessive stress. Move systematically from one side to the other, from the top to the bottom of the well, whilst taking care not to pipette over the same area excessively. After doing this 4–5 times, you should observe that most cells have dissociated from the well.

50. Transfer the entire solution into a tube filled with cold MACS buffer, as outlined in *Step 45b.*
51. Centrifuge at 200 *g* for 3 min in a swinging-bucket rotor centrifuge for efficient pelleting.

***Note:*** If your swinging-bucket rotor centrifuge does not have adaptors for Eppendorfs, use empty 15 mL Falcon tubes as adaptors, but do not exceed 200 g in centrifugation speed, or your Eppendorf will fall through and get stuck in the 15 mL Falcon.

**Figure 10:**
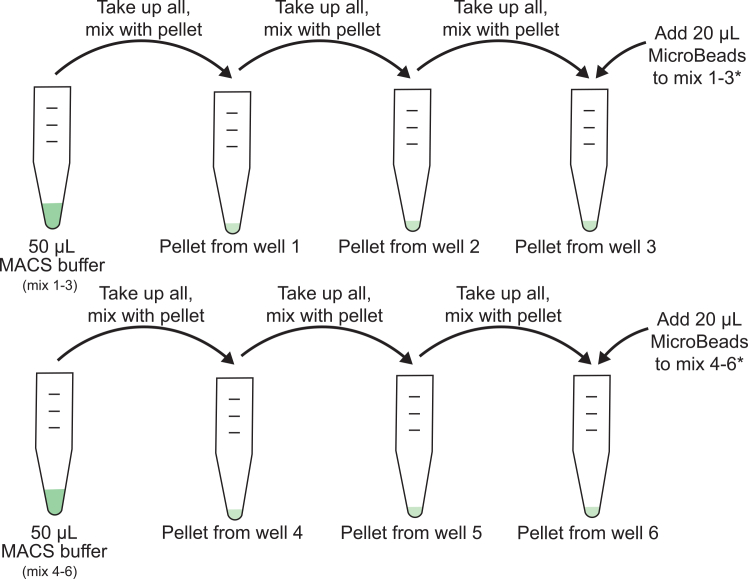
Workflow schematic for combining pellets before the addition of CD144 (VE-cadherin) MicroBeads The workflow corresponds to processing 6 wells of differentiated endothelial cells from the same culture, split across two replicates as outlined in Step 54 and the accompanying Note. The asterisk (∗) relates to the tube layout in Figure 8, specifying the exact tubes used for MicroBeads addition.

***Note:*** At this step, the pellets corresponding to the same sample will be combined into one. For example, if processing three 6-wells, the resulting pellets in three individual 1.5 mL Eppendorfs will now be resuspended and combined into the same 50 μL. As we usually process at least six 6-wells, we generate at least two sets of replicate solutions, each resuspended in 50 μL of MACS buffer (see Figure 10 for a workflow schematic). The combined volume of pellets and 50 μL MACS buffer following resuspension should be approximately 100 μL.

***Note:*** Do not discard the original 1.5 mL Eppendorf tube(s) containing the 50 μL from *Step 45c* and used in *Step 54* – this/these will be used again in *Step 64*.

52. Remove the supernatant and keep the tubes on a cold block.
53. Flick the tubes gently to re-suspend the pellets.
54. Resuspend the pellets in the 50 μL of cold MACS buffer prepared earlier (*Step 45c*; Figure 10).

55. Take the vial of VE-cadherin MicroBeads out of the fridge and swirl to mix.
56. Add 20 μL of the MicroBeads to each cell suspension (approximate volume: 100 μl) with a P20 pipette.
57. Immediately, mix with a P200 pipette by taking the suspension up and down 5 times.
58. Incubate for 15 min in the fridge (4°C).
59. Following incubation, add 1.4 mL of cold MACS buffer to the tube with cells and MicroBeads.
60. Centrifuge at 200 *g* for 3 min in a swinging-bucket rotor centrifuge for efficient pelleting.
61. Aspirate all supernatant, taking care not to disturb the pellet.
62. Flick the tube gently to resuspend the pellet.
63. Add 500 μL of cold MACS buffer and mix gently to combine.
64. Take up the whole cell solution.
  a. Pass through a 40 μm strainer and into the original, sample-specific 1.5 mL Eppendorf tube used for the 50 μL MACS buffer aliquots.
  b. Keep the cell suspension on a cold block.
65. Put the LS column on a magnet.
66. Rinse with 3 mL of cold MACS buffer.
  a. Allow the column to empty fully into the collection tube.

***Note:*** For details on setting up the MACS kit, visit the manufacturer’s website. For example, use the QuadroMACS Kit by Miltenyi Biotec, with set-up photographs available on the website as well.

***Note:*** The number of columns to use per sample will be exactly the same as the number of Eppendorfs prepared with 50 μL MACS buffer in *Step 45c* and, as before, will depend on the initial number of wells used for cell dissociation. For example, for six 6-wells, prepare two LS columns.

67. Once the rinse has passed through, apply each 500 μL cell suspension directly onto the LS column.
  a. Top up with 3 mL of cold MACS buffer.
  b. Allow the column to empty into the collection tube fully.
68. Repeat the 3 mL MACS buffer addition 2 more times for a total of 3 washes.
  a. Allow the column to empty fully between each wash.
69. Carefully remove the column from the separator.
  a. Place it over a 15 mL Falcon tube.
70. Apply 5 mL MACS buffer to the column and push through using the supplied plunger.

***Optional:*** Alternatively, allow the MACS buffer to empty into the new collection tube on its own. This is beneficial when processing multiple samples at once.

71. Count the purified cells to determine the required volume for resuspension at *Step 74.*
72. Once counted, centrifuge the cells at 200 *g* for 3 min in a swinging bucket rotor centrifuge for more efficient pelleting.
73. After centrifugation, remove the supernatant.
74. Resuspend the cells in the required amount of EGM-2 Complete Medium.

***Note:*** We recommend seeding a minimum of 250,000 cells per 12-well (1 mL) and ideally up to 500,000 if possible. The higher cell number will result in confluence upon seeding, which is beneficial for endothelial cell recovery. If seeding 250,000 cells, confluence will be reached within 2 days. Lower seeding numbers are not recommended, especially for ALECs, as these are naturally more quiescent and only proliferate for the first couple of days after MACS. If the cells are seeded too sparsely, they senesce and should not be used for experimentation.

75. Remove the coating from the receiving 12-well plates.
76. Dispense 1 mL of the final cell suspension per well.
  a. Allow to settle in the hood for 10 min before moving to the incubator for a more even cell distribution.

**CRITICAL:** Processing time is extremely important. The removal of laminin coating needs to be followed with cell addition as soon as possible to prevent wells drying out. Ensure that the cell suspension is mixed well before dispensing and repeat the mixing of the source solution after dispensing into four wells to avoid settling of the cells in the stripettes.

### Endothelial cell maturation

**Timing: 5 days (30 min per day)**

Following cell sorting, cells are allowed to undergo maturation to stabilise cell identity and allow recovery from sorting-associated stress. This maturation phase is necessary to ensure robust expression of endothelial markers and functional signalling before downstream applications.

**Figure 11:**
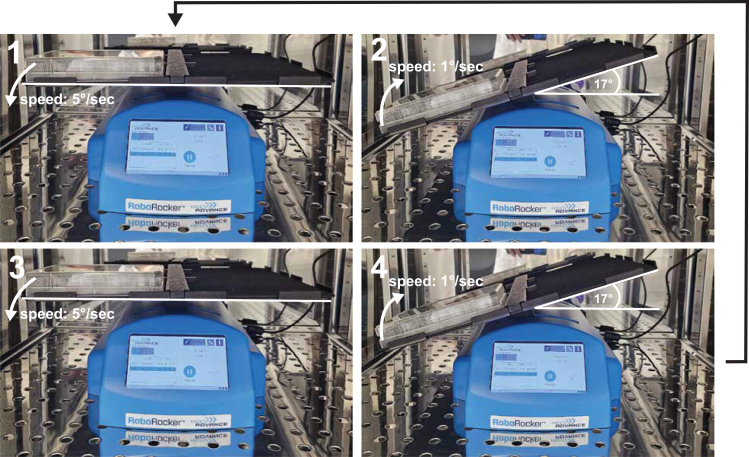
Set-up of RoboRocker 212 in the incubator Still images show the platform at minimum (0°) and maximum (17°) tilt, illustrating the range of rocking motion during the rocking cycle.

***Note:*** We apply flow using a RoboRocker 212 programmed to generate continuous pulsatile (but non-oscillatory) motion, with a tilt rate of 5°/s to a maximum angle of 17°, followed by a return to 0° at 1°/s. We use these rocker settings for ALECs and VLECs cultured in 12-, 24-, and 96-well plates; they are not appropriate for 6-well plates, however, as the resulting medium redistribution will expose cells to drying.

***Note:*** For a 12-well plate, these parameters correspond to an estimated shear stress of 2 × 10^−3^ Pa at the dish center during the forward (fast) stroke. This was calculated using a previously developed model^18^ for calculating fluid shear stress in a culture dish placed on a “see-saw” rocking platform. GitHub code enabling use of this model is available (https://github.com/amorison/rocker-stress); the user can calculate shear stress differences as a function of circular dish diameter, medium volume, viscosity, and rocking pattern.

77. Following 16–24 h of incubation, refresh the EGM-2 Complete Medium.
78. Subject the pure cells to pulsatile (non-oscillatory) flow using a RoboRocker 212 placed inside the incubator (Figure 11).

**Alternatives:** If a RoboRocker 212 is not available, other “see-saw” rocking systems may also be used, subject to the specification of settings that result in equivalent flow to ours. The generated endothelial cells can also be cultured under static conditions. However, we note that their phenotypes are likely to drift faster, requiring careful quality control and experimental interpretation.

79. Repeat for 4 more days, for a total of 5 days of maturation.

***Note:*** We strongly recommend replenishing the medium every day to minimise evaporation; this is crucial under flow as evaporation is accelerated. As with the iPSCs, we recommend consistent daily timings of feeding. After media changes, plates should be returned to the rocker promptly to avoid disrupting flow conditions.

**CRITICAL:** Ensure that the plates are always returned on the rocker in the same orientation to maintain unidirectional flow exposure.

For downstream applications, endothelial cells can be used from 2 days post-MACS onwards, allowing time for recovery, to study endothelial cells at less mature stages.

### Endothelial cell handling for downstream applications

**Timing: 5 days (30 min per day)**

**Figure 12:**
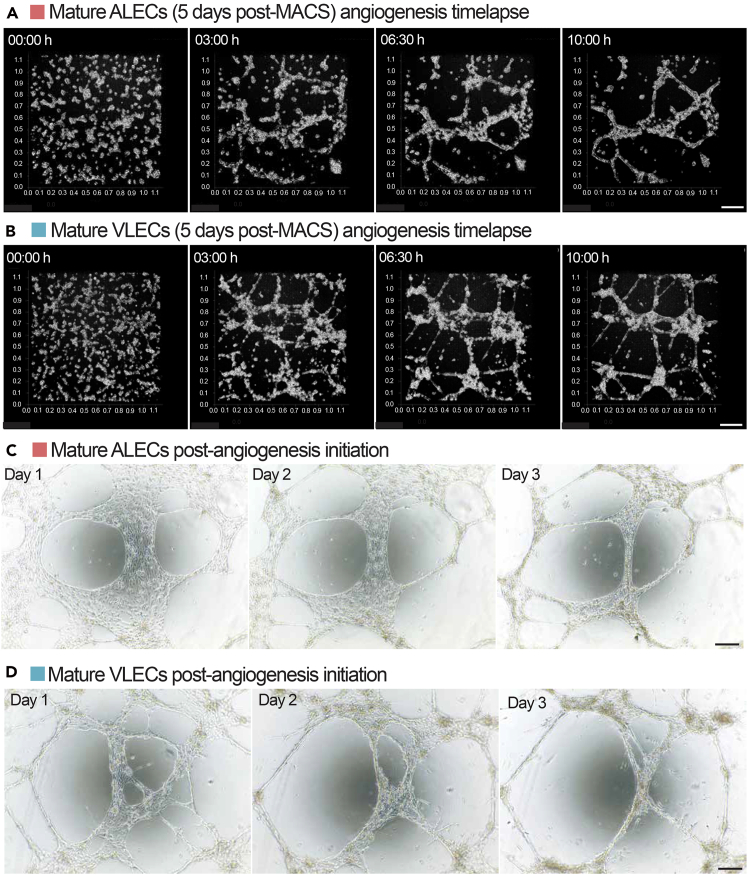
In vitro tube formation assay using iPSC-derived arterial- (ALECs) and venous-like endothelial cells (VLECs) Still images of hourly recordings of mature ALECs (A) or VLECs (B) seeded at 20,000 cells in a 96-well coated with Geltrex (Geltrex Flex LDEV-Free Reduced Growth Factor Basement Membrane Matrix, Thermo Fisher Scientific Cat# A4000046702). The cells were processed for seeding 5 days post-MACS. Still images were generated from brightfield z-stack confocal live-cell imaging (background subtracted) of the EGFP-tagged, endogenous VE-Cadherin, displayed in the XY plane. Daily brightfield micrographs of tube formation from mature ALECs and VLECs are shown in (C) and (D), respectively. Days refer to days post-seeding for the angiogenesis assay. The data are representative of 2 independent experiments and an additional experiment with 2 independent clones performed using a different matrix (growth factor-reduced Matrigel, Corning Cat# 356231). Scale bar (all images): 200 μm.

This section describes experimental handling and evaluation of the endothelial cells following maturation. Cells generated using this workflow are suitable for downstream functional assays, including *in vitro* angiogenesis. The steps described here are limited to general handling and splitting of endothelial cells in 12-well plates. Downstream functional assays should be performed using established or independently developed workflows. The tube assay examples shown in Figure 12 were generated using the publicly available Thermo Fisher Scientific workflow ‘Endothelial Cell Tube Formation Assay - UK’.

80. Prepare in advance:
  a. The required volume of the EGM-2 Complete Medium appropriate for the endothelial lineage (see differentiation media preparation*, Step 40 and associated Notes*).
  b. A tissue-culture treated plate or vessel of your choosing; check specific downstream workflow for additional coating requirements.
  c. EGM-2 Complete Medium and StemPro Accutase pre-warmed to 19°C–23°C before starting.

***Note:*** The required materials and reagents will vary depending on the downstream application and thus are not specified explicitly.

81. Wash each 12-well to be split with 1 mL of DPBS.
82. Treat with 250 μL StemPro Accutase.
  a. Return to the incubator (37°C) for 3–4 min.
83. Bring the plate with dissociated cells back to the biosafety cabinet.
  a. Open and tilt the plate at a 45° angle.
  b. Using a P1000 pipette, gently take up the StemPro Accutase solution with cells.
  c. Dispense back across the well in a systematic motion that goes from one side to the other, from the top to the bottom of the well, taking care not to pipette over the same area excessively.
  d. After doing this 4–5 times, you should observe that most cells have dissociated from the well.
  e. Collect the entire solution into a sterile tube pre-filled with 3 times the amount of Accutase.

**CRITICAL:** Do not scratch the surface of the plate. Instead, wash the well gently just above to avoid agitating the cells.

84. Process as required (see also immunofluorescence for lineage-specific markers).

***Note:*** Endothelial cells generated using this workflow can be difficult to pellet. We therefore recommend centrifugation at 500 *g* for a minimum of 3 min using a swinging-bucket rotor centrifuge with Eppendorf tube adaptors (or open 15 mL Falcons as suitable alternatives).

### Alternative 3D differentiation format for improved VLEC efficiency

**Timing: 6 days (1**–**2 h per day)**

**Figure 13:**
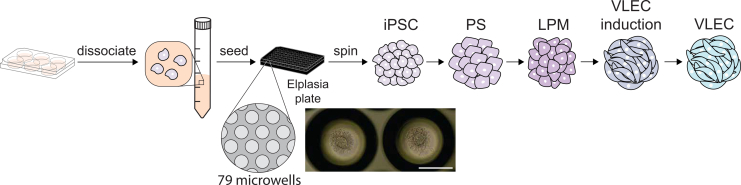
3D differentiation timeline overview iPSC, induced pluripotent stem cell; LPM, lateral plate mesoderm; PS, primitive streak; VLEC, venous-like endothelial cell. A zoomed-in version of a representative microwell is also provided, as indicated by the white rectangle. Scale bar: 250 μm.

Given an overall lower efficiency of VLEC differentiation in 2D, and the need for an additional reseeding step, we also optimised an alternative scaffold-free 3D format for higher efficiency. This version uses identical differentiation solutions as in the main protocol. The key difference is the initial generation of scaffold-free, size-controlled spheroids in ultra-low attachment (ULA) 96-well Elplasia plates containing 79 microwells per well (Figure 13). The following steps detail the modified seeding approach, the key technique for medium exchange, and adaptations required for dissociation of venous spheroids prior to MACS. All other steps, including iPSC maintenance, preparation of differentiation media, and post-MACS endothelial cell maturation, are performed as described in the 2D protocol. The 3D format is recommended for VLEC differentiation; while it can also be applied to ALEC differentiation, it was not further optimised for this purpose and did not support equivalent long-term phenotypic stability compared to the corresponding 2D format.

85. Prepare in advance:
  a. Everything listed under *Experimental seeding of iPSCs Step 1,* except for the ECM-coated 6-well plates.
  b. Manual P200 multichannel pipette.
  c. Integra 300 μL 8 Channel VIAFLO electronic multichannel pipette.
  d. Sterile 25 mL reservoirs.
  e. A 96-well Elplasia plate pre-filled with the required volume of StemFlex Medium supplemented with RevitaCell.
    ***Note:*** This volume will vary depending on the size of spheroids to be seeded, which, in turn, depends on the experimental design. See Table 3 for indicative volumes.
  f. Spin down the pre-filled plate at 500 g for 1 min, then transfer to the incubator.
86. Proceed with *Steps 2*–*6* as described under *Experimental seeding of iPSCs.*

***Note:*** Due to the high-throughput nature of the spheroid protocol, multiple iPSC clones are often seeded within a single 96-well plate. For this, we recommend staggering the dissociation of each line to ensure consistent timing and minimise the risk of mix-ups. For example, while one line is dissociating in Accutase in the incubator, begin washing the wells for the next line with DPBS. Do not leave iPSCs in Accutase for extended periods after dissociation. The number of wells processed per line will depend on the total number of wells to be seeded and the desired spheroid size (please refer to Table 3 with spheroid size/volumes).

87. Centrifuge at 200 *g* for 3 min in a swinging-bucket rotor centrifuge.
88. Remove the supernatant, leaving approximately 50–100 μL of cell suspension behind.
  a. Take care not to disturb the pellet.
89. Flick the tube gently to resuspend the pellet.
90. Resuspend in the required volume of StemFlex Medium supplemented with RevitaCell.

***Note:*** The resuspension volume will depend on the number of wells used for dissociation. It is important to ensure a cell concentration of at least 790,000 cells/mL prior to counting. As a guide, if starting from a sub-confluent iPSC well in a 6-well plate, we recommend resuspending the pellet in 2 mL of medium.

***Note:*** For the 3D set-up, cell counting is performed after centrifugation, in contrast to the 2D workflow. This ensures accurate final cell numbers for consistent spheroid sizing.

91. Take 20 μL of the cell suspension.
  a. Add it to the Eppendorf tube containing Trypan Blue, outlined in *Step 1f.*
  b. Mix thoroughly, then load 10 μL into a haemocytometer. Count the cells manually.
92. Calculate the volume of medium to add to the cells to generate a cell suspension of 790,000 cells/mL.
93. Bring the pre-filled Elplasia plate to the biosafety cabinet.
94. Gently mix the final cell suspension.
  a. Add it to the sterile reservoir for seeding with a multichannel.

**CRITICAL:** The cell suspension must be well-mixed immediately before seeding. To reduce stress on the cells and to ensure consistency, we recommend using the Integra multichannel pipette, with dispense speed set to 5.

95. Immediately aspirate the required volume of cell suspension using the multichannel pipette.
96. Lower the pipette tips into the pre-filled Elplasia wells and dispense the cell suspension (speed 5).

**CRITICAL:** Do not tilt or move the plate at any point during the seeding procedure as this will result in uneven spheroid formation.

97. Once seeding has been completed, spin the Elplasia plate down at 100 g for 1 min to facilitate aggregation prior to returning to the incubator.

**Figure 14:**
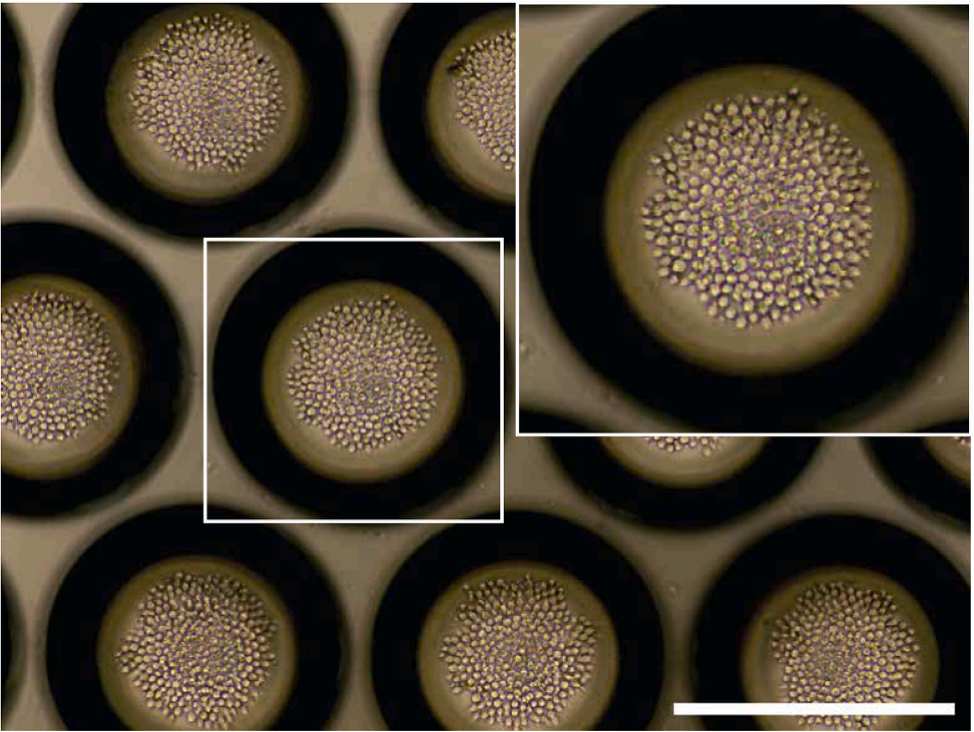
Appearance of the iPSCs immediately following seeding into Elplasia plates at 400 cells/spheroid Scale bar: 500 μm. A zoomed-in version of a representative microwell is also provided, as indicated by the white rectangle.

***Note:*** We recommend checking the seeding under the microscope to confirm consistency across wells and microwells. For an example of the expected appearance, refer to Figure 14.

**Figure 15:**
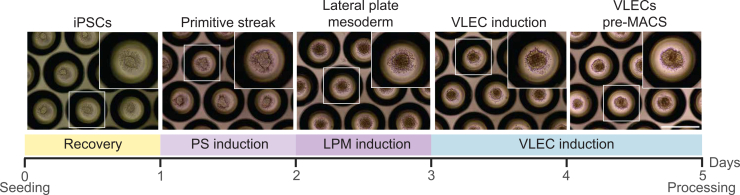
Expected spheroid size and appearance at each differentiation stage A zoomed-in version of a representative microwell from each differentiation stage is also provided, as indicated by the white rectangles. Scale bar: 500 μm.

98. Proceed with VLEC differentiation as described in section lineage-specific induction*.*
  a. Example images of the expected spheroid appearance are shown in Figure 15.

**CRITICAL:** During medium exchange, handle spheroids gently to minimise the risk of dislodgement. This is partially mitigated by the use of the 96-well plate Elplasia format, as opposed to any of the larger Elplasia plate formats. We recommend aspirating medium using a manual multichannel pipette, positioning the tip along the side of the well and gradually lowering it as the medium level decreases. When adding wash or differentiation medium, use the electronic multichannel pipette to enable controlled, gentle dispensing: first add 50 μL per well, followed by a top-up of 150 μL. This approach prevents spheroid drying and minimises variability in differentiation timing across wells. For reference, we use the Integra electronic multichannel pipette set to dispense speed 2.

99. Proceed with MACS as outlined in *Magnetic-activated cell sorting (MACS) for CD144 (VE-cadherin) to enrich for endothelial cell populations,* except for the following:
  a. Wash each 96-well with 200 μL of DPBS, following the same aspiration/dispensing instructions outlined above.
  b. Add 100 μL of StemPro Accutase to each well.
    i. Incubate for 15–20 min in the incubator.
      ***Note:*** The prolonged incubation period is necessary to fully dissociate spheroids into single cells. Shorter incubation times result in incomplete dissociation and cell clumping.
  c. Place a sterile reservoir on a cold block.
  d. Fill the reservoir with the required volume of cold MACS buffer.
    **CRITICAL:** Placing the reservoir on the cold block is essential to keep the MACS buffer chilled throughout cell collection.
    ***Note:*** The volume of MACS buffer needed for collection will vary depending on the number of wells being processed. As a minimum, we recommend adding a volume of MACS buffer equal to the total volume of dissociated cell suspension (e.g., for one 96-well plate with 100 μL of Accutase per well and a total volume of 9.6 mL, add to 10 mL of cold MACS buffer).
  e. Verify under the microscope that the spheroids have dissociated fully into single cells as indicated by the loss of sharp, defined edges.
  f. Using standard P200 tips and a manual multichannel pipette set to 90 μL, pipette up-and-down within each well to aid dissociation.
    ***Note:*** Setting the pipette volume to 90 μL minimizes the formation of bubbles and the associated cell stress.
  g. Dispense the resulting cell suspension into the reservoir containing the cold MACS buffer.
  h. Repeat this process across all remaining wells.
  i. Using the multichannel pipette, pass back across each column of the plate to collect any remaining cell suspension and dispense into the cold MACS buffer.
    ***Note:*** Avoid excess pipetting at this stage to minimize cell stress.
  j. Distribute the collected cell suspension equally across multiple 5 mL Eppendorf tubes (alternatively, 15 mL Falcon tubes can also be used).
  k. Continue with the remainder of the MACS protocol from *Step 51.*

**Table 3:** Volumes of medium and cell suspension to seed in individual Elplasia wells for specific spheroid sizes

Spheroid size (cells/spheroid)	Volume of StemFlex Medium supplemented with RevitaCell to prefill wells (μL)	Volume of 790,000 cells/mL suspension to seed (μL)
100	190	10
200	180	20
300	170	30
400	160	40
500	150	50
1000	100	100

The final volume in each well will be 200 μL.

### Immunofluorescence for lineage-specific markers

**Timing: 1.5 days (4 h hands-on)**

**Figure 16:**
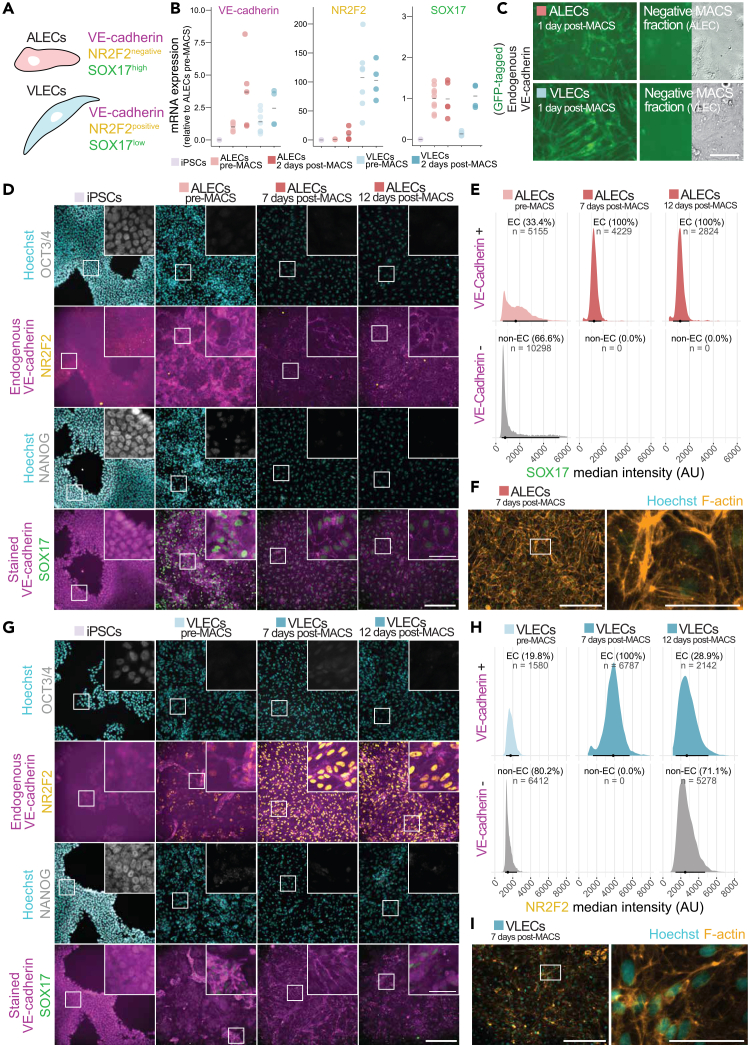
Evaluation of lineage specificity in arterial- (ALECs) and venous-like endothelial cells (VLECs) generated from 2D differentiation of the parental WTC11 EGFP-tagged VE-cadherin iPSCs (A) Schematic of the expected core lineage marker signature for each endothelial subtype.^19^^,^^20^ (B) RT-qPCR assessment of lineage-specific mRNA marker expression in ALECs and VLECs pre- and post-MACS (magnetic-activated cell sorting). The data are representative of a minimum of 2 independent iPSC clones and 4 independent experiments, except for ALECs post-MACS (3 experiments) and VLECs post-MACS (1 experiment). (C) Endogenous EGFP-VE-cadherin expression in ALECs, VLECs, and the corresponding flow-through fractions (negative control), captured by epifluorescence. Brightfield images are also included for the flow-through to visualise the non-endothelial morphology of the negative cell fraction. Scale bar: 50 μm. (D) Undifferentiated (OCT3/4, NANOG), arterial (SOX17), and venous (NR2F2) marker expression in iPSCs and ALECs across the differentiation time-course, measured using immunofluorescence. Scale bar for insets: 50 μm. Scale bar for main micrographs: 200 μm. (E) Single-cell quantification of SOX17 nuclear expression in all cells (top) at the indicated ALEC differentiation stage, or specifically in endothelial (VE-cadherinpos, middle) versus non-endothelial cells (VE-cadherinneg, bottom). The indicated proportion of ECs (33.4%) at the pre-MACS stage was also corroborated by independent cell counting of the subsequent MACS fractions (37.5% VE-cadherinpos cells). (F) F-actin expression in mature ALECs. Scale bar for inset: 50 μm. Scale bar for main micrographs: 200 μm. (G) As in (D) in the context of VLEC differentiation. (H) Single-cell quantification of NR2F2 nuclear expression in all cells (top) at the indicated VLEC differentiation stage, or specifically in endothelial (VE-cadherinpos, middle) versus non-endothelial cells (VE-cadherinneg, bottom). (I) F-actin expression in mature VLECs. Scale bar for inset: 50 μm. Scale bar for main micrographs: 200 μm. The indicated proportion of ECs (33.4%) at the pre-MACS stage was also corroborated by independent cell counting of the subsequent MACS fractions (19.4% VE-cadherinpos cells). Histograms in (E) and (F) show single-cell distributions; median and interquartile range (IQR) are indicated by the horizontal bar underneath each histogram. Nuclei were segmented with StarDist,^21^ median SOX17 or NR2F2 intensity was measured per nucleus, and nuclei lacking VE-cadherin association were manually excluded to obtain VE-cadherin–positive and –negative cell populations. The data are representative of 3 independent clones derived from WTC11 and 3 independent experimental replicates. Full staining panels, including additional markers, across independent differentiation time course experiments are available in Figures S1 (S1) and Figure 2 (S2).

**Figure 17:**
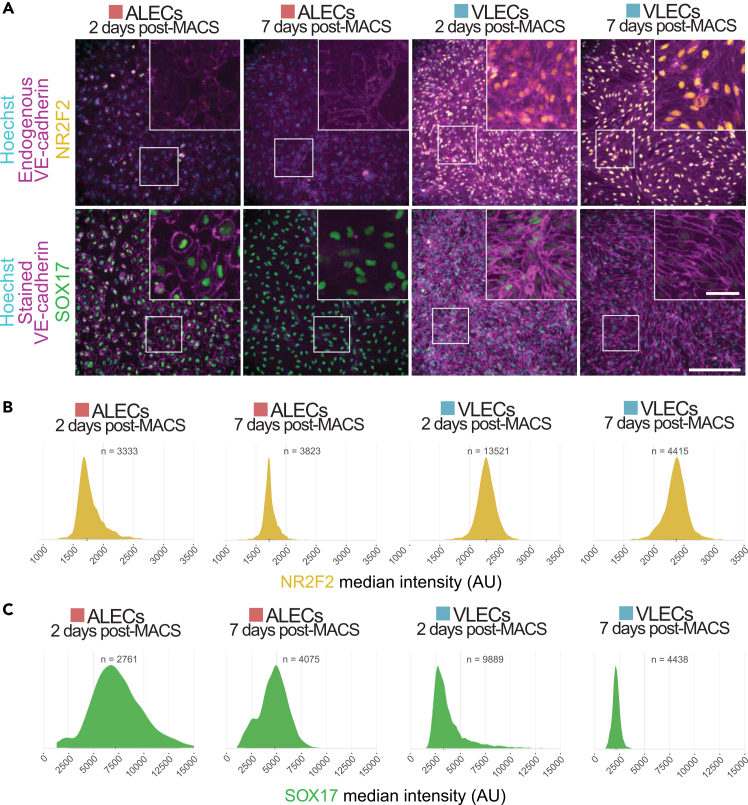
Assessment of lineage maintenance in arterial- (ALECs) and venous-like endothelial cells (VLECs) following 3D differentiation and post-MACS culture (A) Immunofluorescence analysis of venous (NR2F2) and arterial (SOX17) marker expression in ALECs and VLECs at 2 and 7 days post-MACS, derived from the parental EGFP-VE-Cadherin WTC11 iPSC line. Cells were stained for Hoechst (nuclei), endogenous VE-cadherin, and either NR2F2 or SOX17. ALECs are shown for comparative reference; however, we recommend the 2D differentiation workflow for ALEC generation. Insets highlight representative regions; Hoechst staining is omitted in the inset panels to facilitate visualisation of lineage-specific markers. Scale bar for insets: 50 μm. Scale bar for main micrographs: 200 μm. (B) Single-cell quantification of NR2F2 nuclear intensity in ALECs and VLECs at 2 and 7 days post-MACS. Histograms show the distribution of median nuclear intensity values across all segmented cells. (C) Single-cell quantification of SOX17 nuclear intensity in ALECs and VLECs at 2 and 7 days post-MACS. Histograms show the distribution of median nuclear intensity values across all segmented cells. Histograms in (B) and (C) represent single-cell distributions; median and interquartile range (IQR) are indicated by the horizontal bar underneath each distribution. Initial optimisation data for the 3D differentiation workflow, including differentiation efficiency across spheroid sizes measured by flow cytometry of EGFP-tagged VE-cadherin, are provided in Figure S3 (S3). Full staining panels, including additional markers, are available in Figure S4.

**Figure 18:**
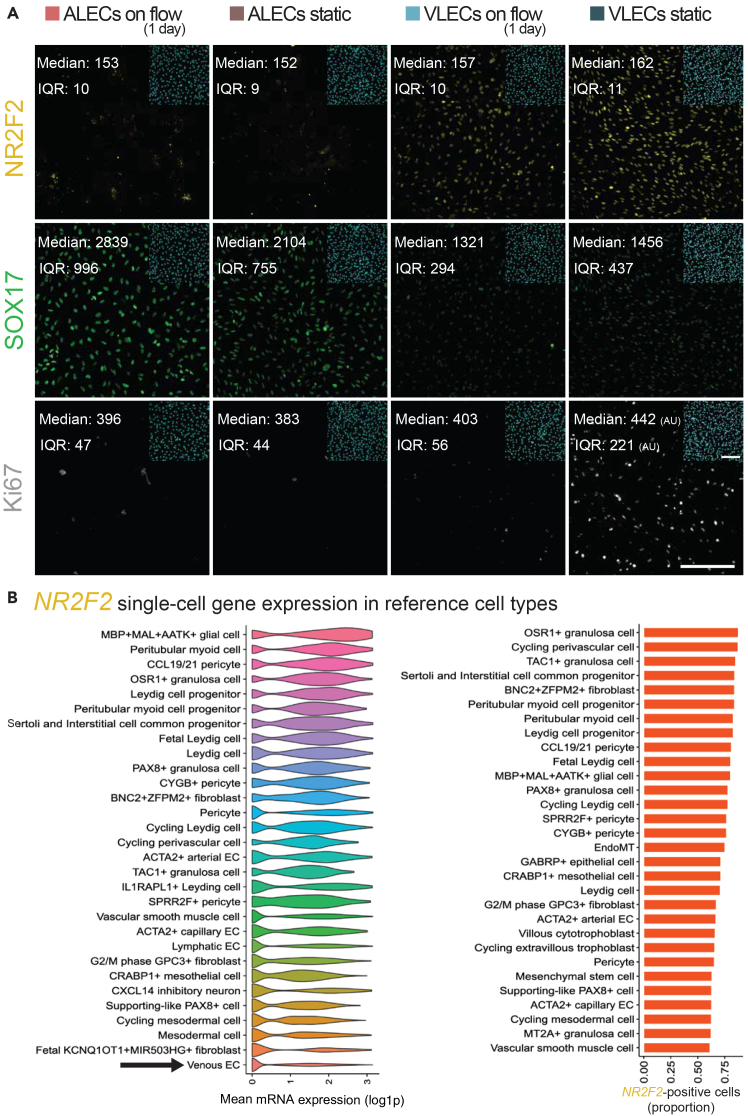
Lineage marker expression in ALECs and VLECs cultured under static versus flow conditions (A) All cultures were assessed 2 days post-MACS, with flow exposure for 24 h before assessment where relevant. ALECs and VLECs were generated from parental WTC11 iPSCs, seeded at 15,000 cells per 96-well (coated with ECMatrix-511 Silk E8 Laminin Substrate). Top panel: expression of the venous NR2F2 marker is low or at background levels in ALECs under both static and flow conditions. In VLECs, NR2F2 expression is higher, with a modest increase observed in static conditions, in line with a concomitant surge in proliferative index as indicated by the extent of Ki67-positive cells (bottom panel). Middle panel: SOX17 expression is enriched in ALECs under flow and reduced under static conditions. Conversely, VLECs show higher SOX17 expression under static culture, indicating emerging loss of lineage fidelity and potential acquisition of an aberrant, mixed arteriovenous phenotype. Insets show Hoechst staining for nuclear identification. Median and interquartile (IQR) staining intensity values for each marker represent quantification of a minimum of 9000 single cells across 9 independent fields of view. The data are representative of 2 independent clones. Scale bar: 200 μm. (B) *NR2F2* mRNA expression across annotated human cell types visualised using DISCO (https://www.immunesinglecell.com/genepage/NR2F2), a public resource compiling previously published single-cell RNA-sequencing datasets.^22^ Histograms represent the top 30 *NR2F2*-expressing lineages by mean gene expression per cell (log-normalised); barplots show the top 30 *NR2F2*-expressing lineages by proportion of positive cells.

This section describes the immunofluorescence (IF) protocol used throughout this workflow to assess pluripotency loss, acquisition of lineage-specific markers, and long-term lineage maintenance in iPSC-derived ALECs and VLECs (representative outputs in Figures 16, 17, and 18). The protocol is optimized for fixed-cell staining in a 96-well plate format using a high-content imaging-compatible vessel (ViewPlate-96 TC black or PhenoPlate-96 TC black; see key resources table) and an automated plate washer.

***Note:*** We recommend reserving at least one column of wells per plate for single-primary and single-secondary antibody-only controls.

***Note:*** All blocking, permeabilization, antibody, and counterstain solutions are prepared at 5X working concentration, because the residual volume left in each well after automated aspiration is approximately 70 μL; addition of 17.5 μL of 5X stock therefore yields the correct 1X final concentration.

100. Prepare in advance:
  a. Cold DPBS for washing.
  b. 4X Fixative: 16% methanol-free formaldehyde stock.
  c. 1X PBS from 10X PBS stock (Severn Biotech; refer to key resources table).
  d. Wash solution (PBS/T): 1X PBS supplemented with 0.05% (v/v) Tween-20. This is also the diluent for permeabilization, blocking, antibody, and counterstain solutions.
  e. 5X permeabilization solution: 0.5% (v/v) Triton X-100 in PBS/T (1:200 dilution from a Triton X-100 stock).
  f. 5X blocking and antibody dilution buffer (5X FSG/PBS/T): 1:2 dilution of the 10X fish skin gelatin (FSG) blocking agent stock in PBS/T.
  g. Plate washer (e.g., Agilent 405TSUV-N or equivalent), with the dispense pin position adjusted so that solutions are added gently against the side of the well, and aspiration height set to leave approximately 70 μL of residual volume per well.

***Note:*** All automated washes are performed as 10 sequential cycles of 100 μL PBS/T or PBS (final wash), dispensed gently against the well wall and aspirated immediately, with no incubation between cycles (Gentle Wash Program or equivalent).

**Alternatives:** All wash steps can also be performed manually if care is taken to ensure gentle dispensing and aspiration.

101. Remove the spent medium gently.
102. Using an electronic multichannel programmed for gentle dispensing, wash all wells with 100 μl cold DPBS.

***Note:*** We use an Integra VIAFLO multichannel set to dispense speed 3.

103. Gently remove the wash.
104. Add 75 μL cold DPBS per well.
105. Fix the cells by adding 25 μL of 16% methanol-free formaldehyde directly to the well to achieve a final concentration of 4% formaldehyde.
106. Incubate for 15 min at 19°C–23°C, protected from light and without shaking.

***Note:*** All fixation steps must be performed in a fume hood using high-grade, methanol-free formaldehyde.

107. After 15 min, remove the fixative.
108. Add 100 μL of DPBS per well.

***Optional:*** Fixed plates can be sealed with Parafilm and stored at 4°C in DPBS for up to 3 months before proceeding.

109. Wash the wells post-fixation by running the automated plate washer program (10 cycles, 100 μL PBS/T per cycle, Gentle Wash Program).

**CRITICAL:** Verify that approximately 70 μL of residual volume remains in each well.

110. Permeabilize the cells by adding 17.5 μL of 5X permeabilization solution to each well.
111. Incubate for 15 min at 19°C–23°C, without shaking.
112. Repeat the automated wash program as in *Step 109.*
113. Block the cells by adding 17.5 μL of 5X blocking solution (5X FSG/PBS/T) to each well.
114. Incubate for 1 h at 19°C–23°C, without shaking.
115. Repeat the automated wash program as in *Step 109.*
116. Prepare each primary antibody solution at 5X working concentration in 5X FSG/PBS/T on a cold block.

**CRITICAL:** Briefly centrifuge each antibody vial before preparing single and multiplexed solutions.

**CRITICAL:** Protect antibody solutions from light if directly conjugated fluorophores are involved.

**CRITICAL:** The recommended final antibody concentrations for the lineage panel used to generate Figures 16, 17, and 18 are listed in the key resources table.

**CRITICAL:** If multiplexing requires use of both unconjugated and fluorophore-conjugated primary antibodies from the same species (e.g., both rabbit), exclude the fluorophore-conjugated primary antibodies from the initial staining solution. Proceed until *Step 124*, then repeat *Steps 116–120* with fluorophore-conjugated primary antibodies, followed by *Step 125* until completion.

117. Aspirate 30 μL from each well to be stained, leaving 40 μL of PBS/T behind.
118. Add 10 μL of the appropriate 5X antibody solution per well.
119. Seal the plate with Parafilm and incubate 16–18 h at 4°C, protected from light.
120. Perform post-primary antibody washes on the plate washer as specified in *Step 109.*
121. Prepare each species-matched secondary antibody solution at 5X working concentration in 5X FSG/PBS/T.

***Note:*** This corresponds to a 1:200 dilution from a 2 mg/mL antibody stock (see also key resources table). For multiplexed conditions, all required secondaries are combined into a single cocktail at the same final concentration.

**CRITICAL:** From this step onward, all incubations and handling must be performed under reduced ambient light to preserve fluorophore intensity. Use highly cross-adsorbed secondary antibodies (see key resources table) to minimize inter-species crosstalk in multiplexed panels.

122. Add 17.5 μL of the 5X secondary solution per well for a final 1:1000 dilution (final concentration 2 μg/mL).
123. Incubate for 1.5 h at 19°C–23°C, protected from light and without shaking.
124. Perform post-secondary antibody washes on the plate washer as specified in *Step 109.*
125. Prepare Hoechst 33342 at 5X working concentration (1:200 dilution in PBS/T).
126. Add 17.5 μL per well for a final 1:1000 dilution.
127. Incubate for 15 min at 19°C–23°C, protected from light and without shaking.

***Note:*** If co-staining with Phalloidin (for F-actin), combine both Hoechst and Phalloidin into a 5X stock in PBS/T, then aspirate 30 μL to leave 40 μL of residual volume per well, and add 10 μL of the 5X concentrated working solution(s) for a final 1:5 dilution. Extend the incubation to 30 min.

128. Run the automated wash program (see *Step 109*) using 1X PBS only (no Tween-20).

**CRITICAL:** Removal of Tween-20 in the final wash is important to minimize the formation of refractive particulates that interfere with high-content imaging.

***Optional:*** Stained plates can be sealed with Parafilm and stored in the dark at 4°C until imaging — typically the following day, but for up to two weeks without appreciable signal loss.

129. Image acquisition. Image the plate on a high-content confocal imager. The data presented in this protocol were acquired on an Operetta CLS system (PerkinElmer) using a 20X (0.4 NA) objective, with 9 fields of view (3 × 3 non-overlapping grid centered at the geometric center of a well) collected per well.

## Expected outcomes

This workflow results in ALECs and VLECs, as defined by loss of undifferentiated markers and acquisition of lineage-specific markers (for 2D: Figure 16; Figures S1 and S2; for 3D; Figure 17; Figures S3 and S4). Consistent with prior observations in human arteries and veins, our iPSC-derived ALECs also exhibit prominent F-actin stress fibres (Figure 16F) in contrast to a largely cortical F-actin distribution in VLECs (Figure 16I).^23^ As a minimum, when assessing differentiation success, we recommend immunofluorescence for VE-cadherin, SOX17, and NR2F2 as the most robust and reproducible readout of differentiation efficiency, which also allows for confirmation of correct localisation. By contrast, flow cytometry-based analysis is not localisation-specific and can be confounded by subjective gating strategies and inadvertent exclusion of off-target populations based on cell size, an issue noted in some prior studies where the full gating strategy was made available. We therefore recommend a two-pronged approach. For example, we used flow cytometry during optimisation of the 3D protocol to evaluate endogenous EGFP-tagged VE-cadherin expression, but this was followed by unbiased, high-content imaging-based validation of the purified cells using our standard immunofluorescence protocol.

With the current implementation, 2D differentiation efficiencies are moderate, ranging from 20%–60% for VLECs and 30%–70% for ALECs prior to MACS. These estimates are derived from immunofluorescence-based quantification and are corroborated by manual counts of VE-cadherin-positive and -negative fractions following MACS. The reported ranges reflect variability across multiple iPSC lines, including male and female lines, CRISPR-edited lines, and patient-derived lines carrying *PIK3CA* mutations. They are also reproducible across independent operators (n=4). In side-by-side comparisons, ALEC differentiation yield is consistently higher than VLEC differentiation under 2D conditions, which is also consistent with the original PI3K inhibitor-based protocol.^10^ It is possible that lower differentiation efficiencies reflect our use of CRISPR-edited iPSC lines, including the parental EGFP-tagged VE-Cadherin WTC11.

Following MACS, ALECs retain their identity for at least 12 days (Figure 16). In contrast, VLECs maintain identity during the first week post-MACS, after which progressive dedifferentiation is observed, with venous endothelial cells comprising approximately 29% of the population by day 12 (Figure 16). This pattern is reversed in 3D, where VLEC differentiation efficiencies of approximately 80% or higher are typically achieved (Figure 17; Figure S3). Moreover, VLECs generated in 3D retain their identity for at least one week post-MACS, whereas the corresponding ALECs show loss of phenotypic stability within the first week (Figure 17). The underlying reasons for these differences are not fully defined yet, but may reflect variations in cell-autonomous extracellular matrix production in the scaffold-free format. In addition to improving VLEC differentiation efficiency, the 3D workflow for both ALECs and VLECs is compatible with single-cell signaling analyses, including mass cytometry, as described previously.^24^ For long-term culture and maturation, we recommend the 2D format for ALEC generation and the 3D format for VLEC generation.

We note that purified ALECs and VLECs must be maintained at confluence to preserve their lineage identity, consistent with the confluent organisation of endothelial cells in mature vessels *in vivo*. In contrast, sparse seeding results in rapid senescence and aberrant cellular morphology. Lastly, we note that for studies investigating the developmental consequences of genetic PI3K activation, intermediate differentiation efficiencies can be advantageous, as they provide sufficient dynamic range to detect both positive and negative lineage enrichments.

## Limitations

### Differentiation yield

Differentiation efficiency is sensitive to the quality of the starting iPSC cultures. Reduced pluripotency, diminished viability, or suboptimal culture conditions prior to differentiation initiation will lead to variable and inconsistent outcomes. In addition, we have found that the iPSC culture density before processing for single-cell seeding can influence subsequent proliferation dynamics, confluence, and thus overall differentiation efficiency. Differentiation efficiency can also vary as a function of iPSC line, especially if they are engineered to harbour pathological mutations. In 3D, the differentiation efficiency varies with spheroid size and thus initial seeding density. We recommend 300–500 cells/spheroid at the start of 3D differentiation, with 400 cells/spheroid as the optimal number. We recommend the VE-cadherin MACS step to ensure consistently pure populations of ALECs and VLECs. However, a caveat of MACS is the loss of cells during processing and thus a reduction in the final yield of differentiated cells.

Different culture workflows and coating substrates exist for iPSCs and will impact differentiation efficiency. In this workflow, we opted for commercially available and fully defined reagents, namely StemFlex and ECMatrix-511 Silk E8 Laminin Substrate. We also tested the workflow using iPSCs cultured in Essential 8 Flex Medium (Thermo Fisher Scientific #A2858501) on plates coated with 10 μg/cm^2^ Cultrex Stem Cell Qualified Reduced Growth Factor Basement Membrane (R&D Systems #3434-010-02). When using this culture format, we noted consistently poorer survival following single-cell seeding and removal of RevitaCell 24 h later, which compromised the robustness of subsequent endothelial cell differentiation.

It remains unclear why arterial differentiation in 3D, but not in 2D, results in reduced phenotypic stability following the VE-cadherin MACS step, despite identical downstream culture conditions. Given the critical role of NOTCH signaling in arterial specification and maintenance,^25^ it is possible that the 3D differentiation format does not support sufficient activation or maintenance of this pathway. Further work will be required to resolve this mechanistically.

### Long-term maintenance and maturation

In general, ALECs and VLECs generated and maintained according to this workflow should not be kept for more than 7 days post-MACS, as they may begin to lose their phenotype, especially if subjected to repeated passaging. We routinely assess lineage stability by immunofluorescence of spare cells seeded in 96-well plates at the time when the main cultures are used for experiments, and recommend that others do the same to ascertain continuous lineage purity. We have consistently noted improved long-term lineage stability when the cells are exposed to pulsatile, albeit gentle (and thus non-physiological) flow on the RoboRocker 212 (Figure 18A). Although culture under static conditions leads to a modest increase in NR2F2 expression in VLECs, it is also accompanied by a dramatic increase in Ki67-positive nuclei and a partial increase in SOX17 expression, suggesting an aberrant, mixed-lineage identity. Furthermore, NR2F2 is widely used to distinguish venous from arterial endothelial cells; however, it is also highly expressed in many non-endothelial cell lineages (Figure 18B).

It is possible that further improvements in lineage stability and thus long-term culture can be achieved by exposure to physiologically relevant arterial and venous shear stress levels. Nevertheless, one should bear in mind that endothelial cells and ALECs, in particular, are quiescent as expected physiologically, and successful long-term maintenance without artificial immortalisation is unlikely.

The endothelial culture medium used here post-MACS contains 2% fetal bovine serum; alternatively, serum-free differentiation strategies have been described^9^^,^^26^ and could be incorporated in future optimisations to ensure fully xeno-free culture conditions post-differentiation.

Generally, iPSC-derived endothelial cells present with an immature phenotype, with further maturation taking place upon exposure to flow. In the context of PI3K-driven vascular disorders, the more immature nature of the cells could be advantageous as it may better recapitulate developmental processes.

## Troubleshooting

### Problem 1: Poor differentiation efficiency

Low differentiation efficiency (*Steps 45*–*71)* may result from multiple factors, including variability in starting iPSC quality, culture density, suboptimal culture conditions before differentiation initiation (see details in daily iPSC maintenance) as well as extensive genetic engineering of the iPSC lines. While some variability in differentiation efficiency is expected, consistently achieving efficiencies below 20% should prompt troubleshooting.

### Potential solution

• Ensuring high quality of the starting iPSC culture. Initiating experiments with low-quality or partially differentiated cells can compromise downstream results. Maintaining cell health is therefore critical and can be supported by using freshly prepared culture medium, in which supplements are mixed into the base medium shortly prior to use. This approach helps preserve buffering capacity and nutrient stability. In our experience, cells show improved performance when cultured in freshly prepared medium, and aliquoting supplemented medium is an effective strategy to maintain media integrity and consistency over time. Additionally, we recommend iPSC incubators not to be in close proximity to centrifuges, as the resulting vibrations can compromise culture performance.
• Minimize extensive genetic engineering. Genetic engineering of iPSCs can result in bottleneck selection and enrichment of private genetic variants that may compromise cell health and differentiation performance. Repeated rounds of genetic engineering and subclonal selection should therefore be minimized wherever practically possible.
• Ensuring optimal seeding density. Initial seeding density is critical for efficient differentiation. In our experience, cultures seeded too sparsely show poor viability and reduced differentiation efficiency, and the cells generally perform better when plated at higher density (i.e., with sufficient cell–cell contact). This is particularly important for arterial differentiation, where a substantial proportion of cells may be lost during commitment to the arterial lineage; starting with an adequate density helps maintain cell health and supports robust differentiation outcomes. In addition, following MACS, it is important to maximize cell–cell contact; not doing so can lead to rapid senescence and a marked decline in cell quality. This finding aligns with previous reports demonstrating that higher seeding density and increased cell–cell contact promote optimal differentiation and functional properties in iPSC-derived brain microvascular endothelial cells.^27^
• Taking note of the geometry of the well. We observe that differentiation efficiency and the extent and timing of cell death can vary between plate formats (e.g., 6-well vs 96-well). This likely reflects format-dependent differences in the local culture microenvironment, including media height (volume-to-surface area), gas and nutrient exchange, dilution or accumulation of cell-secreted factors, and evaporation or edge effects (particularly in 96-well plates). While we do not systematically optimise for all plate formats, we recommend that users be aware of these effects and consider plate geometry, vessel size, and manufacturer when comparing results or transferring the workflow across different culture formats.
• Ensuring high quality of differentiation reagents. Careful tracking of differentiation reagents and their expiry is essential. Reported stability windows may differ depending on formulation, storage conditions, and handling, and users are encouraged to validate reagent stability themselves where feasible. For instance, despite reported instability in water, we have successfully used NKH477 (forskolin analogue) for extended periods at 4°C without detectable degradation, as verified by NMR. Users should independently confirm stability under their own conditions.
• Monitoring temperature or CO_2_ fluctuations. Differentiation efficiency is highly sensitive to temperature and CO_2_ stability. Even small or transient deviations from set conditions can result in pronounced suboptimal cell health and reductions in differentiation efficiency. For example, we have observed dramatic losses in differentiation performance during periods of incubator instability. Users should therefore minimise incubator door openings, limit the time cells spend outside the incubator, and closely monitor incubator temperature, CO_2_, and humidity levels throughout the differentiation process.

### Problem 2: Inaccurate VE-cadherin evaluation

Antibody-based VE-cadherin staining (*Steps 100*–*129*) should be interpreted with caution and evaluated in conjunction with cellular morphology and protein localization, not solely based on the presence of a signal. Correct VE-cadherin localization is characterized by sharp, continuous appearance at cell-cell junctions, producing well-defined membrane borders, as observed in endothelial cells (example inset in Figure 16). In contrast, although signal is also detectable in iPSCs (example inset in Figure 16), the staining pattern is diffuse and distributed throughout the cell rather than restricted to the membrane border, reflecting non-specific antibody binding as confirmed by the lack of endogenous signal from the EGFP-tagged VE-cadherin allele.

Detection of VE-cadherin may also be influenced by antibody accessibility and epitope availability. We have consistently observed that in arterial-like endothelial cells, the antibody-based VE-cadherin signal is often weaker relative to the endogenous VE-cadherin reporter, whereas in venous endothelial cells, antibody staining can appear comparatively stronger. This likely reflects the tighter intercellular junctions in arterial cells, leading to steric hindrance or reduced epitope accessibility. Notably, we have observed that antibody-based detection of VE-cadherin is more variable across samples, in contrast to detection of the EGFP-tagged endogenous protein. Additionally, in cells with weak VE-cadherin expression, the antibody may fail to detect a signal altogether, as observed in 2D VLECs 12 days post-MACS (Figure 16G).

### Potential solution

Tagging endogenous VE-cadherin with a fluorescent reporter provides a more reliable readout of its expression and junctional localisation compared to antibody-based staining alone.

## Resource availability

### Lead contact

Further information and requests for resources and reagents should be directed to and will be fulfilled by the lead contact, Dr. Ralitsa R. Madsen (ralitsa.madsen@glasgow.ac.uk).

### Technical contact

Technical questions on executing this protocol should be directed to and will be answered by the technical contact, Ms. Oliwia N. Mruk (3213760m@student.gla.ac.uk).

### Materials availability

This study did not generate new, unique reagents.

### Data and code availability

• Microscopy data reported in this paper will be shared by the lead contact upon request.
• This paper does not report original code. Any additional information required to reproduce the data reported in this paper is available from the lead contact upon request.

## Acknowledgments

This work was funded by the CLOVES Syndrome Community (R.R.M.), the CARAVAN Network (R.R.M.), 10.13039/100004440Wellcome (Sir Henry Wellcome Fellowship 220464/Z/20/Z to R.R.M.; PhD studentship 218520/Z/19/Z to A.J.M.), 10.13039/100014013UKRI (Future Leaders Fellowship MR/Y017439/1 to R.R.M.), and 10.13039/501100000265MRC (PhD studentship to O.N.M., MRC PPU core funding grant MC_UU_00038).

We would like to thank Professor Robert Semple for helpful discussions and Bruce Conklin for sharing the parental WTC11 iPSC line with us. We thank Miss Sweta Swaminathan for her help with brightfield image acquisition of iPSC maintenance cultures. We thank all members of the Madsen Lab for support with iPSC maintenance, data discussions, and final proofreading of the manuscript. We are grateful to Dr. Adrien Morison, University of Glasgow, for his help with developing the Python script for shear stress calculations. Lastly, we are indebted to the University of Dundee’s Imaging Facility (DIF), the National Phenotypic Screening Centre (NPSC), as well as the MRC PPU Lab Management and Technical Teams. Finally, we acknowledge Ang et al. and Loh et al. and their respective teams for establishing the original ALEC/VLEC differentiation protocols, upon which this work builds. Their prior efforts and documentation were essential to the success of the present study.

## Author contributions

Conceptualisation, R.R.M.; methodology, O.N.M., A.J.M., and R.R.M.; data curation, O.N.M., A.J.M., and R.R.M.; formal analysis, O.N.M., A.J.M., and R.R.M.; investigation, O.N.M., A.J.M., and R.R.M.; project administration, R.R.M.; resources, R.R.M.; supervision, R.R.M.; validation, O.N.M., A.J.M., and R.R.M.; visualisation, O.N.M., A.J.M., and R.R.M.; writing – original draft, O.N.M. and R.R.M.; writing – review and editing, O.N.M., A.J.M., and R.R.M.

## Declaration of interests

R.R.M. has received consulting fees from Nested Therapeutics (Cambridge, U.S.) and serves on the Scientific Advisory Board of CLOVES Syndrome Community.

## Declaration of generative AI and AI-assisted technologies in the writing process

During the preparation of this work, the authors used ChatGPT (OpenAI) and Claude (Anthropic) for assistance with textual revisions. After using this tool/service, the authors reviewed and edited the content as needed and take full responsibility for the content of the published article.

## References

[1] Castillo S.D., Baselga E., Graupera M. (2019). PIK3CA mutations in vascular malformations. Curr. Opin. Hematol..

[2] Kurek K.C., Luks V.L., Ayturk U.M., Alomari A.I., Fishman S.J., Spencer S.A., Mulliken J.B., Bowen M.E., Yamamoto G.L., Kozakewich H.P.W., Warman M.L. (2012). Somatic Mosaic Activating Mutations in PIK3CA Cause CLOVES Syndrome. Am. J. Hum. Genet..

[3] Sterba M., Pokorna P., Faberova R., Pinkova B., Skotakova J., Seehofnerova A., Blatny J., Janigova L., Koskova O., Palova H. (2023). Targeted treatment of severe vascular malformations harboring PIK3CA and TEK mutations with alpelisib is highly effective with limited toxicity. Sci. Rep..

[4] Green T.E., Garza D., Brown N.J., de Silva M.G., Bennett M.F., Tubb C., Phillips R.J., MacGregor D., Robertson S.J., Bekhor P. (2024). Improving genetic diagnostic yield in a large cohort of children with rare vascular anomalies or PIK3CA-related overgrowth spectrum. Genet. Med. Open.

[5] Tan W.-H., Baris H.N., Burrows P.E., Robson C.D., Alomari A.I., Mulliken J.B., Fishman S.J., Irons M.B. (2007). The spectrum of vascular anomalies in patients with PTEN mutations: implications for diagnosis and management. J. Med. Genet..

[6] Gurunathan A., Ricci K., Iacobas I., Rednam S.P., Wusik K., Fei L., Hammilll A.M. (2020). Impact of vascular anomalies on the PTEN phenotype in children and young adults. Pediatr. Blood Cancer.

[7] Dykman M., Pillai N.R., Lenhart K., Nicholson C., Boull C., Fritz E., Flanagan S., Maguiness S. (2024). Arteriovenous malformations as a presenting sign of PTEN hamartoma tumor syndrome: A case series. Pediatr. Dermatol..

[8] Abdelilah-Seyfried S., Ola R. (2024). Shear stress and pathophysiological PI3K involvement in vascular malformations. J. Clin. Investig..

[9] Sriram G., Tan J.Y., Islam I., Rufaihah A.J., Cao T. (2015). Efficient differentiation of human embryonic stem cells to arterial and venous endothelial cells under feeder- and serum-free conditions. Stem Cell Res. Ther..

[10] Ang L.T., Nguyen A.T., Liu K.J., Chen A., Xiong X., Curtis M., Martin R.M., Raftry B.C., Ng C.Y., Vogel U. (2022). Generating human artery and vein cells from pluripotent stem cells highlights the arterial tropism of Nipah and Hendra viruses. Cell.

[11] Pan Z., Yao Q., Kong W., Ma X., Tian L., Zhao Y., Zhu S., Chen S., Sun M., Liu J. (2025). Generation of iPSC-derived human venous endothelial cells for the modeling of vascular malformations and drug discovery. Cell Stem Cell.

[12] Ang L.T., Zheng S.L., Liu K.J., Masaltseva A., Winters J., Creytz I. von, Jha S.K., Yin Q., Qian C., Xiong X. (2025). Discovery of a pre-vein progenitor that requires VEGF/ERK inhibition to complete vein differentiation. bioRxiv.

[13] Loh K.M., Zheng S.L., Liu K.J., Yin Q., Amir-Ugokwe Z.A., Jha S.K., Qi Y., Wazny V.K., Nguyen A.T., Chen A. (2025). Protocol for efficient generation of human artery and vein endothelial cells from pluripotent stem cells. STAR Protoc..

[14] Miyazaki T., Futaki S., Suemori H., Taniguchi Y., Yamada M., Kawasaki M., Hayashi M., Kumagai H., Nakatsuji N., Sekiguchi K., Kawase E. (2012). Laminin E8 fragments support efficient adhesion and expansion of dissociated human pluripotent stem cells. Nat. Commun..

[15] Nguyen M.T.X., Okina E., Chai X., Tan K.H., Hovatta O., Ghosh S., Tryggvason K. (2016). Differentiation of Human Embryonic Stem Cells to Endothelial Progenitor Cells on Laminins in Defined and Xeno-free Systems. Stem Cell Rep..

[16] Song J., Zhang X., Buscher K., Wang Y., Wang H., Di Russo J., Li L., Lütke-Enking S., Zarbock A., Stadtmann A. (2017). Endothelial Basement Membrane Laminin 511 Contributes to Endothelial Junctional Tightness and Thereby Inhibits Leukocyte Transmigration. Cell Rep..

[17] Ohgushi M., Matsumura M., Eiraku M., Murakami K., Aramaki T., Nishiyama A., Muguruma K., Nakano T., Suga H., Ueno M. (2010). Molecular pathway and cell state responsible for dissociation-induced apoptosis in human pluripotent stem cells. Cell Stem Cell.

[18] Zhou X., Liu D., You L., Wang L. (2010). Quantifying fluid shear stress in a rocking culture dish. J. Biomech..

[19] Sissaoui S., Yu J., Yan A., Li R., Yukselen O., Kucukural A., Zhu L.J., Lawson N.D. (2020). Genomic Characterization of Endothelial Enhancers Reveals a Multifunctional Role for NR2F2 in Regulation of Arteriovenous Gene Expression. Circ. Res..

[20] Corada M., Orsenigo F., Morini M.F., Pitulescu M.E., Bhat G., Nyqvist D., Breviario F., Conti V., Briot A., Iruela-Arispe M.L. (2013). Sox17 is indispensable for acquisition and maintenance of arterial identity. Nat. Commun..

[21] Schmidt U., Weigert M., Broaddus C., Myers G., Frangi A.F., Schnabel J.A., Davatzikos C., Alberola-López C., Fichtinger G. (2018). Medical Image Computing and Computer Assisted Intervention – MICCAI 2018.

[22] Li M., Ang K.S., Teo B., Rom U., Nguyen M.N., Maurer-Stroh S., Chen J. (2025). Rediscovering publicly available single-cell data with the DISCO platform. Nucleic Acids Res..

[23] van Geemen D., Smeets M.W.J., van Stalborch A.-M.D., Woerdeman L.A.E., Daemen M.J.A.P., Hordijk P.L., Huveneers S. (2014). F-Actin–Anchored Focal Adhesions Distinguish Endothelial Phenotypes of Human Arteries and Veins. Arterioscler. Thromb. Vasc. Biol..

[24] Madsen R.R., Le Marois A., Mruk O.N., Voliotis M., Yin S., Sufi J., Qin X., Zhao S.J., Gorczynska J., Morelli D. (2025). Oncogenic PIK3CA corrupts growth factor signaling specificity. Mol. Syst. Biol..

[25] Fish J.E., Wythe J.D. (2015). The Molecular Regulation of Arteriovenous Specification and Maintenance. Dev. Dyn..

[26] Rosa S., Praça C., Pitrez P.R., Gouveia P.J., Aranguren X.L., Ricotti L., Ferreira L.S. (2019). Functional characterization of iPSC-derived arterial- and venous-like endothelial cells. Sci. Rep..

[27] Wilson H.K., Canfield S.G., Hjortness M.K., Palecek S.P., Shusta E.V. (2015). Exploring the effects of cell seeding density on the differentiation of human pluripotent stem cells to brain microvascular endothelial cells. Fluids Barriers CNS.

