# Supplementary material for: Protocol for PI3K inhibitor-free differentiation and maturation of human iPSC-derived arterial- and venous-like endothelial cells
Source: STAR Protoc. 2026 Jul 30;7(3):104668. doi: 10.1016/j.xpro.2026.104668 (PMC13452351; doi:10.1016/j.xpro.2026.104668)
Supplement: Supplementary file 1 [file mmc1.pdf]

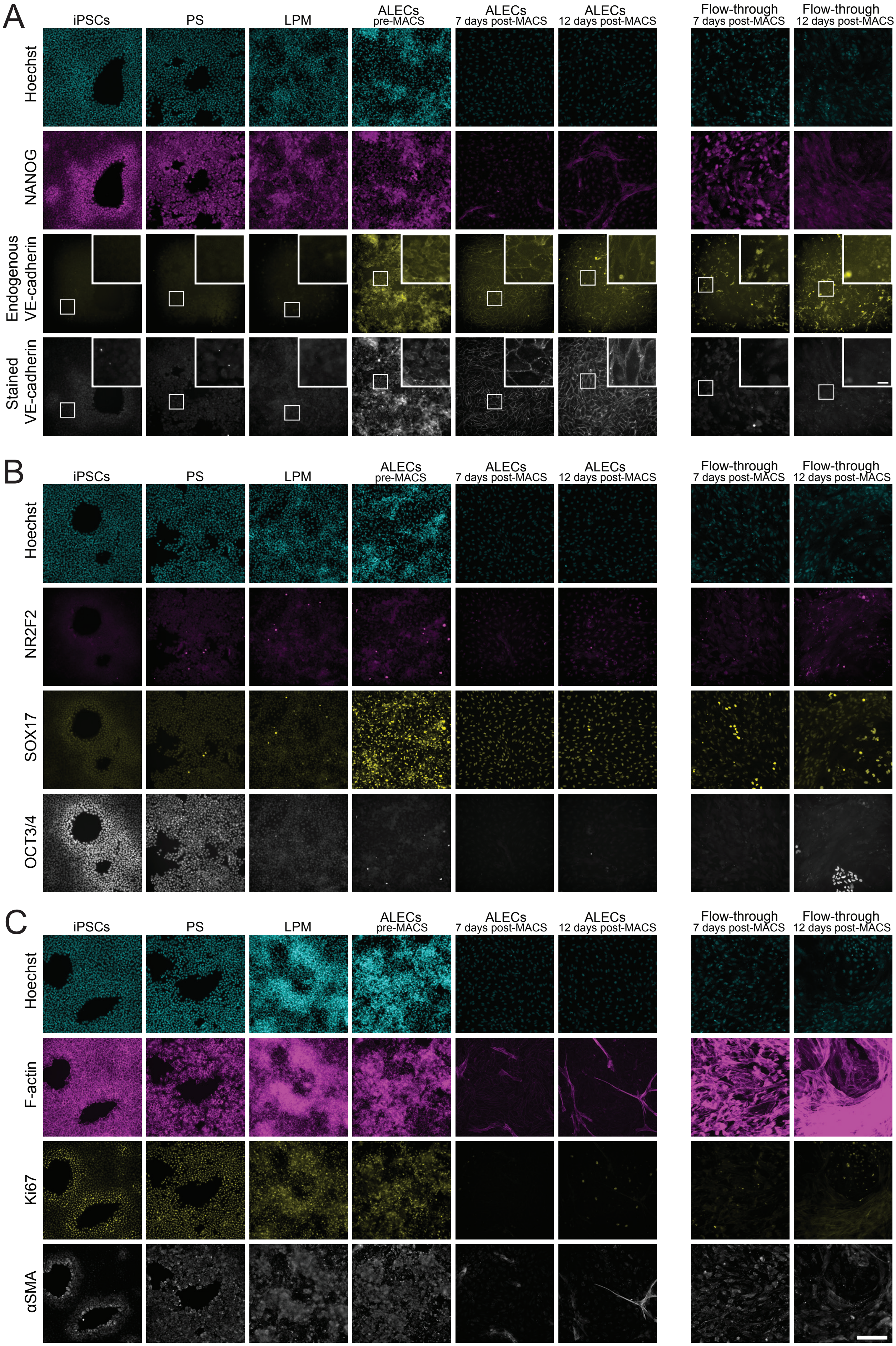
A
B
C
iPSCs
PS
LPM
ALECs pre-MACS
ALECs 7 days post-MACS
ALECs 12 days post-MACS
Flow-through 7 days post-MACS
Flow-through 12 days post-MACS
Hoechst
NANOG
Endogenous VE-cadherin
Stained VE-cadherin
NR2F2
SOX17
OCT3/4
F-actin
Ki67
αSMA

Supplementary Figure 1: Full staining panel for the differentiation timeline of WTC11 EGFP-tagged VE-cadherin iPSCs to mature arterial-like endothelial cells (ALECs). A. NANOG (undifferentiated marker) and VE-cadherin (endothelial marker) expression at individual stages of iPSC-derived ALEC differentiation, measured by immunofluorescence. VE-cadherin expression in differentiated ALECs is further validated by the fluorescent signal and junctional localization of the endogenous, EGFP-tagged protein. **B.** Immunofluorescence for OCT3/4 (undifferentiated marker), NR2F2 (venous marker) and SOX17 (arterial marker in the context of ECs) throughout ALEC differentiation, confirming the expected OCT3/4^{neg} NR2F2^{neg} SOX17^{high} ALEC signature. **C.** Immunofluorescence for F-actin, Ki67 and αSMA throughout ALEC differentiation. Consistent with their arterial phenotype, pure ALEC cultures present with emerging F-actin-rich tube-like structures and minimal expression for Ki67 and αSMA. All panels also include images from the corresponding MACS flow-through fractions (negative controls), alongside Hoechst staining for cell identification. The data are representative of differentiations from 3 independent clones and 3 experimental replicates, with pure ALECs kept on flow. Scale bar for insets: 20 µm. Scale bar for main micrographs: 200 µm. Abbreviations: ALECs: arterial-like endothelial cells; αSMA: α-smooth muscle actin; iPSCs: induced pluripotent stem cells; LPM: lateral plate mesoderm; MACS: magnetic-activated cell sorting; PS: primitive streak.

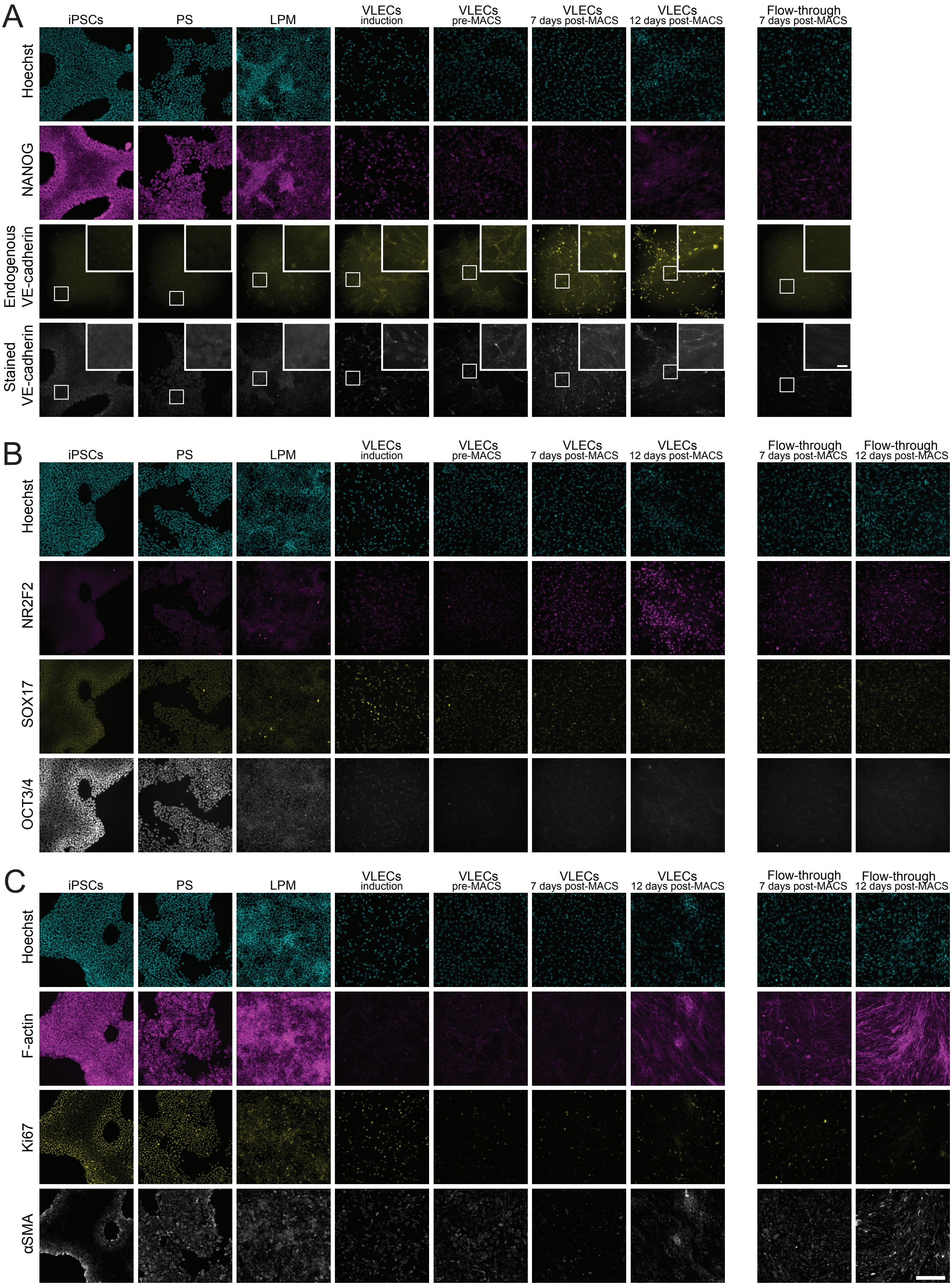
A
iPSCs
PS
LPM
VLECs induction
VLECs pre-MACS
VLECs 7 days post-MACS
VLECs 12 days post-MACS
Flow-through 7 days post-MACS
Hoechst
NANOG
Endogenous VE-cadherin
Stained VE-cadherin
B
iPSCs
PS
LPM
VLECs induction
VLECs pre-MACS
VLECs 7 days post-MACS
VLECs 12 days post-MACS
Flow-through 7 days post-MACS
Flow-through 12 days post-MACS
Hoechst
NR2F2
SOX17
OCT3/4
C
iPSCs
PS
LPM
VLECs induction
VLECs pre-MACS
VLECs 7 days post-MACS
VLECs 12 days post-MACS
Flow-through 7 days post-MACS
Flow-through 12 days post-MACS
Hoechst
F-actin
Ki67
αSMA

Supplementary Figure 2: Full staining panel for the differentiation timeline of WTC11 EGFP-tagged VE-cadherin iPSCs to mature venous-like endothelial cells (VLECs). A. NANOG (undifferentiated marker) and VE-cadherin (endothelial marker) expression at individual stages of iPSC-derived VLEC differentiation, measured by immunofluorescence. VE-cadherin expression in differentiated VLECs is further validated by the fluorescent signal and junctional localization of the endogenous, EGFP-tagged protein. **B.** Immunofluorescence for OCT3/4 (undifferentiated marker), NR2F2 (venous marker) and SOX17 (arterial marker in the context of ECs) throughout VLEC differentiation, confirming the expected OCT3/4^{neg} NR2F2^{pos} SOX17^{low} VLEC signature. **C.** Immunofluorescence for F-actin, Ki67 and αSMA throughout ALEC differentiation. Consistent with their arterial phenotype, pure VLEC cultures present with emerging F-actin-rich tube-like structures, moderate expression for Ki67 and minimal αSMA expression. All panels also include images from the corresponding MACS flow-through fractions (negative controls), alongside Hoechst staining for cell identification. The data are representative of differentiations from 3 independent clones and 3 experimental replicates, with pure VLECs kept on flow. Scale bar for insets: 20 µm. Scale bar for main micrographs: 200 µm. Abbreviations: αSMA: α-smooth muscle actin; iPSCs: induced pluripotent stem cells; LPM: lateral plate mesoderm; MACS: magnetic-activated cell sorting; PS: primitive streak; VLECs: venous-like endothelial cells.

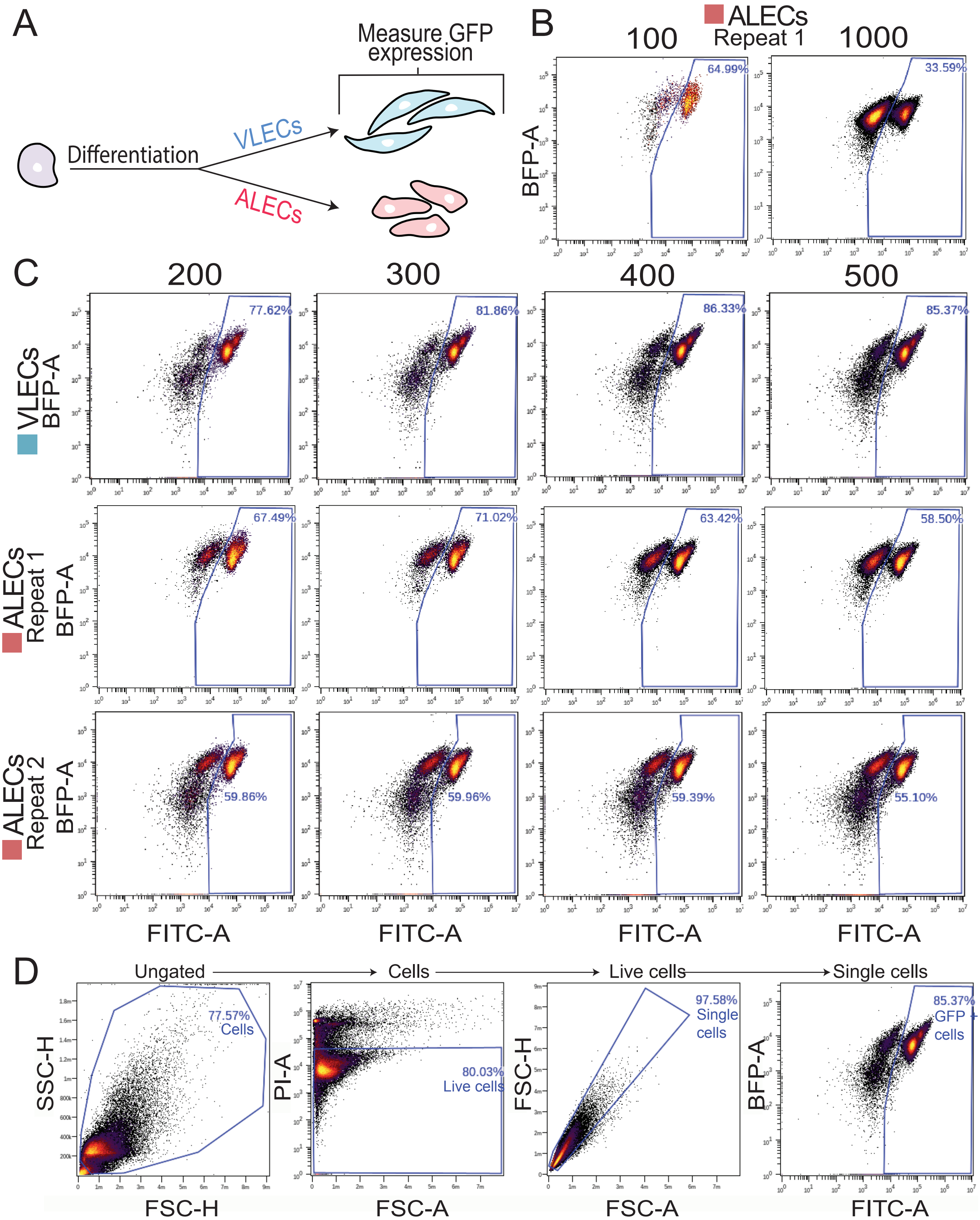

Supplementary Figure 3: Optimisation of the 3D ALEC and VLEC differentiation protocol. A. Schematic overview of the screening strategy used to optimize 3D differentiation conditions. Human EGFP-VE-cadherin WTC11 iPSCs were differentiated into arterial- (ALECs) and venous-like endothelial cells (VLECs) in spheroids of defined size, and endothelial differentiation efficiency was quantified based on EGFP-tagged VE-cadherin expression measured by flow cytometry. **B.** Representative flow cytometry

plots showing ALEC differentiation efficiency for spheroids generated with 100 and 1000 cells per spheroid. Both conditions were suboptimal, with low cell yield at small spheroid size (100 cells) and reduced differentiation efficiency at large spheroid size (1000 cells). **C.** Flow cytometry analysis of EGFP-VE-cadherin expression across increasing spheroid sizes for both VLECs (top panels) and ALECs (bottom panels). Differentiation efficiency is indicated as the percentage of EGFP-positive cells within each condition. Intermediate spheroid sizes yielded the highest efficiency for VLEC differentiation, whereas ALEC differentiation remained comparatively less efficient under 3D conditions. EGFP signal was detected in the FITC-A channel, with the empty BFP-A channel used to aid gating and exclude background signal. **D.** Gating strategy used for analysis in (B) and (C), including sequential selection of cells (FSC/SSC), live cells (PI exclusion), single cells (FSC-A vs FSC-H), and EGFP-positive endothelial cells. Percentages indicate the proportion of events retained at each gating step. The example shown corresponds to the 500 cells/spheroid VLEC condition in (C).

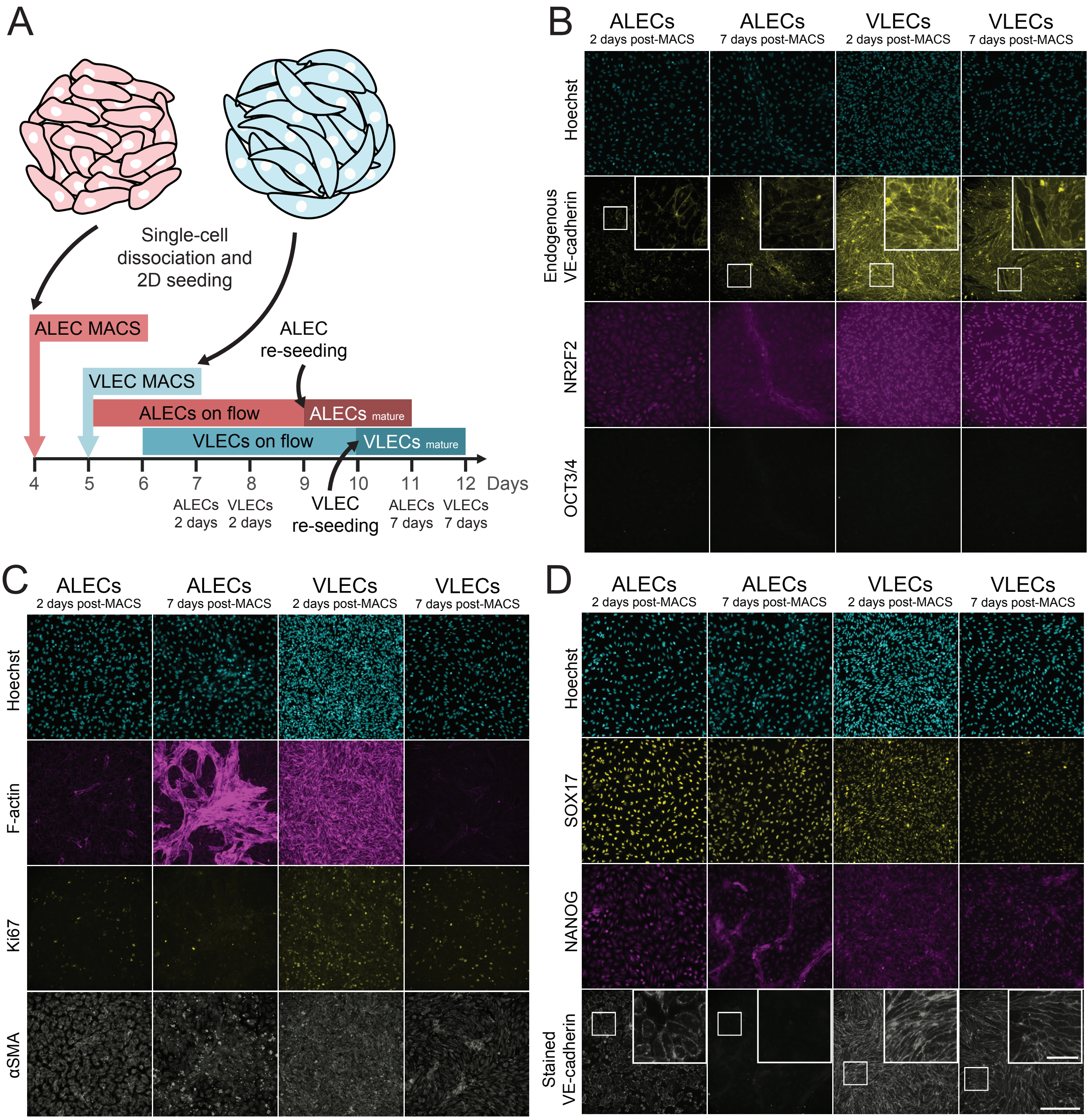

Supplementary Figure 4: Full immunofluorescence staining panels for ALECs and VLECs generated from 3D differentiation of the parental EGFP-VE-cadherin WTC11 iPSC line. A. Schematic of the timeline, including timepoints of dissociation and fixing at 2 and 7 days post-MACS for immunufluorescence staining. **B-D.** Immunofluorescence analysis of VE-cadherin (endothelial marker), NR2F2 (venous marker), OCT3/4 (undifferentiated marker), SOX17 (arterial marker in the context of ECs), NANOG (undifferentiated marker), Ki67 (proliferation marker), F-actin (cytoskeletal marker) and αSMA (mesenchymal marker) in ALECs and VLECs at 2 and 7 days post-MACS. VE-cadherin expression is further validated by the fluorescent signal and junctional localization of the endogenous, EGFP-tagged protein. VLECs derived from the 3D protocol display a stable NR2F2^{high} SOX17^{low} phenotype and cortical F-actin, consistent with venous identity. In contrast, ALECs obtained with the 3D differentiation protocol display disorganised morphology and reduced structural integrity over time. Ki67 and αSMA

expression remain low across conditions, indicating limited proliferation and abscence of mesenchymal off-target differentiation. All panels include Hoechst staining for nuclear identification. The data are representative of two independent experiments using WTC11-derived clones. Scale bar for insets: 50 μm. Scale bar for main micrographs: 200 μm.
